# Predicting Penumbra Salvage and Infarct Growth in Acute Ischemic Stroke: A Multifactor Survival Game

**DOI:** 10.3390/jcm12144561

**Published:** 2023-07-08

**Authors:** Gaia Sirimarco, Davide Strambo, Stefania Nannoni, Julien Labreuche, Carlo Cereda, Vincent Dunet, Francesco Puccinelli, Guillaume Saliou, Reto Meuli, Ashraf Eskandari, Max Wintermark, Patrik Michel

**Affiliations:** 1 Stroke Center, Neurology Service, Department of Clinical Neurosciences, Lausanne University Hospital, University of Lausanne, 1011 Lausanne, Switzerlandpatrik.michel@chuv.ch (P.M.); 2 Neurology Unit, Department of Internal Medicine, Riviera Chablais Hospital, 1847 Rennaz, Switzerland; 3 Statistical Unit, Regional House of Clinical Research, University of Lille, CHU Lille, EA 2694—Santé Publique: Épidémiologie et Qualité des Soins, 59000 Lille, France; 4 Stroke Center, Neurology Service, Ospedale Civico di Lugano, 6900 Lugano, Switzerland; 5 Diagnostic and Interventional Radiology Service, Lausanne University Hospital, University of Lausanne, 1011 Lausanne, Switzerland; 6 Department of Diagnostic and Interventional Radiology, Neuroradiology Division, Stanford University and Medical Center, Stanford, CA 94305, USA

**Keywords:** acute ischemic stroke, penumbra salvage, infarct growth, perfusion computerized tomography, acute stroke treatment

## Abstract

Background. Effective treatment of acute ischemic stroke requires reperfusion of salvageable tissue. We investigated the predictors of penumbra salvage (PS) and infarct growth (IG) in a large cohort of stroke patients. Methods. In the ASTRAL registry from 2003 to 2016, we selected middle cerebral artery strokes <24 h with a high-quality CT angiography and CT perfusion. PS and IG were correlated in multivariate analyses with clinical, biochemical and radiological variables, and with clinical outcomes. Results. Among 4090 patients, 551 were included in the study, 50.8% male, mean age (±SD) 66.3 ± 14.7 years, mean admission NIHSS (±SD 13.3 ± 7.1) and median onset-to-imaging-time (IQR) 170 (102 to 385) minutes. Increased PS was associated with the following: higher BMI and lower WBC; neglect; larger penumbra; absence of early ischemic changes, leukoaraiosis and other territory involvement; and higher clot burden score. Reduced IG was associated with the following: non-smokers; lower glycemia; larger infarct core; absence of early ischemic changes, chronic vascular brain lesions, other territory involvement, extracranial arterial pathology and hyperdense middle cerebral artery sign; and higher clot burden score. When adding subacute variables, recanalization was associated with increased PS and reduced IG, and the absence of haemorrhage with reduced IG. Collateral status was not significantly associated with IG nor with PS. Increased PS and reduced IG correlated with better 3- and 12-month outcomes. Conclusion. In our comprehensive analysis, multiple factors were found to be responsible for PS or IG, the strongest being radiological features. These findings may help to better select patients, particularly for more aggressive or late acute stroke treatment.

## 1. Introduction

The treatment of acute ischemic stroke is actually focused on reperfusion of the penumbra and the salvaging of potentially viable tissue. Positron emission tomography and experimental studies have identified different hypoperfusion regions within the ischemic area at an early stage [1,2,3,4], including irreversibly damaged, at-risk viable tissue, and oligemic tissue without risk [5,6]. The affected tissue changes over time and the conversion of the penumbra into infarction is a dynamic process, with the irreversible damage progressing from the core of ischemia to the periphery. Magnetic resonance imaging (MRI) evaluation of lesions further supports the concept of the variable fate of the penumbra and the possibility of detecting the mismatch of irreversible/reversible damaged tissue [7,8]. Indeed, the penumbra can be accurately estimated using automated thresholding techniques in real time with MRI or with computerized tomography perfusion (CTP). In the DEFUSE and EPITHET studies, “Target Mismatch” patients showed better clinical and radiological outcomes after reperfusion than the “No Target Mismatch” group, probably because of the presence of minimal or no salvageable tissue in these patients [9,10]. While time is an important surrogate for the progression of infarction, there is massive variability in progression within and between individual patients [11], even after revascularization [12]. Two trials, DAWN and DEFUSE-3, have provided new evidence showing the clinical usefulness of a tissue viability mismatch-based selection of patients for thrombectomy in an extended therapeutic time window [13,14]. Similarly, a meta-analysis confirmed a better functional outcome for patients with unknown stroke onset and stroke onset in the late window when treated by intravenous alteplase after salvageable tissue had been identified by neuroimaging [15]. Recent results also support the prognostic role of pre-treatment perfusion parameters for patients with large vessel occlusion treated by EVT in the early window, showing an association between an unfavourable mismatch and a poor three-month functional outcome that is independent of the time from stroke onset [16].

In fact, whereas some patients lose neurons at a high rate, others maintain regions of salvageable ischemic tissue for many hours [17]. Additionally, the mere presence of penumbra does not predict a better outcome by itself, but multiple clinical, biological and radiological determinants influence its fate [18]. These findings support the concept that time-independent variables play an important role in this setting [19], hinting at a complex of intertwined relationships of multiple factors determining penumbra salvage (PS) and infarct growth (IG).

Using retrospective data from a large cohort of acute ischemic stroke patients, we aimed to investigate the predictors of PS and IG among multiple clinical, biological and radiological factors. We also wanted to assess the magnitude of the impact of PS and IG on different clinical long-term outcomes.

## 2. Materials and Methods

### 2.1. Patient Selection

Data from the Acute Stroke Registry and Analysis of Lausanne (ASTRAL) from January 2003 to June 2016 were analysed for this study. ASTRAL is a single-centre prospective cohort of consecutive acute ischemic stroke patients admitted to the stroke centre and/or intensive care unit of the Lausanne University Hospital (CHUV) within 24 h of an ischemic stroke [20]. It incorporates detailed clinical, laboratory data and CT-based multimodal brain imaging techniques to investigate underlying causes and mechanisms of ischemic stroke.

We included patients with acute ischemic stroke involving the middle cerebral artery (MCA) territory with or without ipsilateral anterior cerebral artery (ACA) or posterior cerebral artery (PCA) involvement and last seen well (LSW) less than 24 h. For imaging criteria, we included patients with available high-quality, CT-based imaging that had been performed within 24 h of symptoms onset including CTP. The CTP had to meet the following requirements: hypoperfusion region consistent with acute clinical symptoms; reaching at least established penumbra thresholds; total ischemia volume (core and penumbra) of at least 10 mL; and availability of CTP maps reconstructed with a standardized method in a core lab [21]. A repeated CT or MRI of good quality to assess the final infarct volume was also required to be available. We excluded patients with unknown stroke onset and pre-existing visible infarct in the newly hypoperfused area.

Collection and scientific use of data in ASTRAL were approved by the institution’s ethical commission (Commission d’Ethique de la Recherche Clinique, UNIL, protocol 40/07).

The Strengthening the Reporting of Observational Studies in Epidemiology (STROBE) method was applied [22].

### 2.2. Clinical Variables

Demographic data, medical history and vascular risk factors were recorded. We collected pre-stroke modified Rankin scale (mRs) and current medications at the time of the index event. We recorded neurological symptoms and signs, as well as stroke characteristics at admission and subacute phase (24–48 h after stroke onset). We also measured clinical and biochemical parameters on admission and subacute phase. Stroke aetiology was classified according to the TOAST classification [23], with dissection and multiple causes added as categories.

Reperfusion therapy was defined as intravenous thrombolysis (IVT) and/or endovascular treatment (EVT). IVT was administered in accordance with the European Stroke Organisation [24] and Swiss guidelines at the time of hospitalization [25]. We calculated onset-to-door, onset-to-brain-imaging, and onset-to-treatment times, including onset-to-needle/groin puncture and door-to-needle time.

Clinical outcome was measured at 3 and 12 months using the mRS, either at the outpatient stroke clinic or by standardized telephone interview by Rankin-certified medical staff. Outcome was assessed as the delta-mRS, i.e., the difference between 3-month (12-month) mRS and prestroke mRS. An excellent outcome was considered as 3- and 12-month mRS ≤ 1 and favourable outcome as 3- and 12-month mRS ≤ 2. We also recorded all causes of mortality.

### 2.3. Imaging Protocol

We examined all patients admitted to our institution with suspected acute ischemic stroke with a multimodal CT scan as part of standard of care, unless contrast contraindication existed. One experienced vascular neurologist (PM) and two senior neuroradiologists, all with a 15-year experience in multimodal CT, independently reviewed all images. In case of discordance, a consensus was obtained at regular joint multidisciplinary meetings.

Cerebral CT was performed on a 16-multidetector CT scanner (LightSpeed, GE Healthcare, Milwaukee, WI, USA) until November 2005 and on a 64-multidetector CT scanner (LightSpeed VCT, GE Healthcare, Milwaukee, WI, USA) thereafter. Non-contrast CT (NCCT) and post-contrast series were acquired in axial mode from the skull base to the vertex using the following imaging parameters: 120 kV peak tube voltage, 320 mA tube current, slice thickness 5 mm, 32 cm scan field of view (SFOV), and 512 × 512 matrix. NCCT was performed to detect intracranial haemorrhage, hyperdense middle cerebral artery sign, early haemorrhagic transformation, chronic stroke lesions and the presence of leukoaraiosis. We defined chronic cerebrovascular lesions as the presence of chronic infarct and/or the presence of leukoaraiosis (defined as grade ≥ 1 according to the Blennow scale) [26]. Early ischemic changes (cortico–subcortical dedifferentiation, insular ribbon sign, isodense basal ganglia and/or hypoattenuation) in the MCA territory were recorded to calculate ASPECTS on NCCT [27].

CT angiography (CTA) was acquired in helical scan mode (120 kV peak tube voltage, 150–260 mA tube current, 0.984:1 pitch, 0.625 mm slice thickness, 50 cm SFOV, 512 × 512 matrix) from the aortic arch to the top of the frontal sinuses, after injection of 50 mL of iodinated contrast (Accupaque 300, iohexol 300 mg/mL) at a flow rate of 5 mL per second (delay according to the perfusion data), followed by 50 mL of 0.9% saline solution at the same flow rate. Significant extracranial or intracranial arterial pathology in the ischemic territory, defined as the presence of ≥50% stenosis, occlusion, dissection or floating thrombus was recorded. We calculated clot burden score (CBS) for each patient as an indicator of clot extension [28]. The CBS is a scoring system used to define the extent of thrombus and is scored on a scale of 0–10. A score of 10 is normal, implying clot absence. The collateral score was graded according to Tan et al. [29]. Absence of collateral flow to the ischemic territory was graded as 0, whereas collateral flow in ≤50%, >50% and 100% of the vessels filling in the ischemic territory distal to the occluded artery were graded as 1, 2 and 3, respectively. For statistical analysis, we grouped grades 0 and 1 as ‘poor’ collaterals, and grades 2 and 3 as ‘good’ collaterals.

CTP was performed in patients arriving ≤ 24 h with a suspicion of supratentorial stroke to assess for acute focal hypoperfusion. All CTP series were acquired in axial scan mode with 80 kV peak tube voltage, 100 mA tube current, 32 cm SFOV and 512 × 512 matrix. CTP were positioned at the level of the basal ganglia and the third ventricle above the orbits in order to protect the lens. Four 10-mm slices (40 mm z-axis coverage) were imaged until November 2005 and 16 5-mm slices (80 mm z-axis coverage) were imaged from November 2005 on. CTP images were acquired for 50 s in a cine mode with a delay of 5–7 s after beginning the injection of 50 mL of iodinated contrast (Accupaque 300, iohexol 300 mg/mL, GE Healthcare, Glattbrugg, Switzerland) into an antecubital vein using a power injector at a flow rate of 5 mL per second, followed by 50 mL of 0.9% saline solution at the same flow rate. CTP data were subsequently analysed using the Brilliance Workspace Portal^®^ (Philips Medical Systems, Cleveland, OH, USA), based on the central volume principle using deconvolution to create parametric maps of mean transit time (MTT); cerebral blood volume (CBV) was calculated from the area under the time–enhancement curves and cerebral blood flow (CBF) was derived from the formula CBF = CBV/MTT. We calculated the penumbra and infarct maps as follows: a total ischemic volume (penumbra and infarct) is defined as cerebral pixels with a greater than 145% prolongation of MTT compared with the corresponding region in the contralateral unaffected cerebral hemisphere. In this selected area, 2.0 mL per 100 g was chosen as the rCBV threshold and pixels with rCBV values higher or lower than 2.0 mL per 100 g were attributed, respectively, to the penumbra or the infarct. Lesion surface on the final PCT maps was measured automatically on each of the four slices and the volume of the penumbra and infarct then calculated by multiplication by the slice thickness [21].

A control imaging was performed at approximately 24 h in patients receiving acute recanalization treatment to assess ischemic and haemorrhagic changes. The final infarct volume was measured in a core lab, based on the fluid-attenuated inversion recovery (FLAIR) sequences of the first MRI performed more than 24 h after stroke onset. MRI was performed on a 3-Tesla scanner (Magnetom Vida^®^, Siemens Healthcare, Erlangen, Germany). If FLAIR was not performed, we used the larger of the DWI or T2W volumes. If no subacute MRI was available, the first NCCT performed beyond 24 h after stroke was used. If significant oedema with radiological mass effect was present at the time of the first subacute imaging, a later imaging (MRI if possible, otherwise NCCT) without mass effect was used.

Penumbra salvage was calculated as the difference between the total ischemia volume on baseline CTP and the final infarct volume. Infarct growth was calculated as the difference between the final infarct volume and the core volume on baseline CTP.

Haemorrhagic transformation was classified in radiological and symptomatic groups according to ECASS-II [30]. Parenchymal haemorrhage was defined by the presence of a parenchymal hematoma type 1 or 2 according to ECASS II definition [30].

In patients with arterial occlusion at baseline, recanalization was assessed at 24 h (with a range of 12–48 h) by CTA, or MR-angiography. Recanalization of occluded intracranial arteries was classified as absent and partial (residual stenosis of ≥50%, or <50% but with persistent stenosis or occlusion distal to the initial occlusion site) or complete (residual stenosis < 50%, no distal stenosis or occlusion to the original occlusion site), as published in detail in [31].

### 2.4. Statistical Analysis

We expressed continuous variables as means (standard deviation) or medians (interquartile range, IQR) for non-normal distribution and categorical variables as numbers (percentage). Normality of distributions was assessed using histograms and the Shapiro–Wilk test. Regarding the non-normal distribution of PS and IG without simple transformation, PS and IG were divided into tertiles to assess their associations with acute and subacute characteristics and outcomes.

We investigated the acute and subacute predictors of PS and IG using ordinal logistic regression models adjusted for baseline penumbra volume and infarct core, respectively. Acute characteristics associated with PS with a *p* < 0.10 in baseline penumbra-adjusted (core-adjusted for the IG) analyses were implemented into a first multivariable ordinal logistic model with a backward-stepwise approach using a removal criteria of *p* > 0.10. A second backward multivariable ordinal logistic model including both acute and subacute predictors (with a *p* < 0.10 in baseline penumbra-adjusted analyses) was performed. The absence of collinearity between the candidate variables to multivariable models was checked by calculating the variance inflation factors (VIFs) [32]. We further investigated the acute and subacute predictors of PS and IG using multinomial logistic regression models using the same selection strategy (as a secondary analysis). To avoid case deletion in multivariate analyses due to missing data on acute and subacute characteristics, missing data were imputed by multiple imputations using a regression-switching approach (chained equations with m = 10). An imputation procedure [33] was performed under the missing-at-random assumption using all acute and subacute characteristics (including penumbra salvage and infarct growth) with a predictive mean-matching method for continuous variables and logistic regression models (binary, ordinal or multinomial) for categorical variables. Estimates obtained from the different imputed data sets were combined using the Rubin’s rules [34]. The same statistical analyses were performed to assess the predictors of IG.

Finally, we assessed the associations of PS and IG with 3- and 12-month outcomes (excellent, favourable, overall degree of disability and all-cause mortality) using binary or ordinal logistic regression models before and after adjustment for independent predictors of PS and IG.

Statistical testing was undertaken at the two-tailed α level of 0.05. Data were analysed using the SAS software package, release 9.4 (SAS Institute, Cary, NC, USA).

## 3. Results

**Figure 1:**
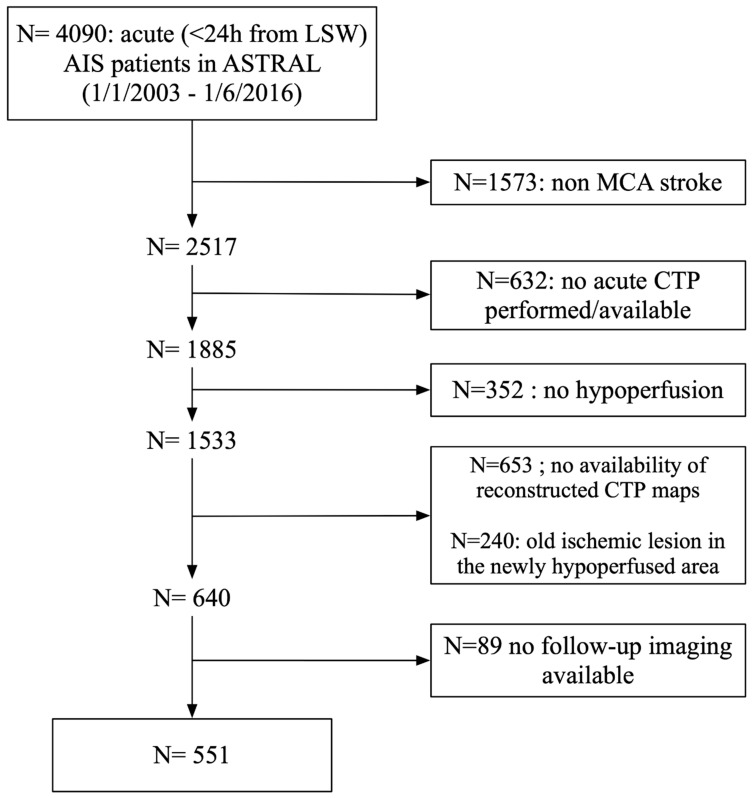
This flow diagram illustrates the selection strategy and inclusion/exclusion criteria used to derive the final study population.

From 2003 to 2016, 4090 consecutive patients were included in ASTRAL, of whom 551 patients with MCA strokes, a high-quality CTA and CTP that met the requisite threshold were included in the present study (Figure 1). We summarize patient characteristics, clinical and radiological findings and outcomes for the study sample in Table 1. Median age of the study cohort was 68.7 years (IQR 21 years), 271 (49.2%) were female and the median baseline NIHSS was 14 points (IQR 12 points). Median time from symptoms onset to CT scan was 170 min (IQR, 102 to 385 min) with a median ASPECTS of 8 (IQR, 6 to 10). At baseline, the median penumbra volume was 63 mL (IQR, 23 to 109) and the median infarct core was 29 mL (IQR, 8 to 76 mL). Among the 273 (49.6%) patients receiving acute revascularization treatments, 256 patients were treated by IVT and 17 patients by EVT. At 24 h after admission, 47.1% of occluded arteries had partial or complete recanalization. The median PS volume was 59 mL (IQR, 16 to 118) and the median IG was 0 mL (IQR, −15 to 18). Favourable outcome rate was 52.8% (95%CI: 48.6–57.1%) at 3 months and 54.9% (95%CI, 50.7 to 59.1%) at 12 months. All-cause mortality occurred in 13.9% (95%CI, 11.0 to 16.8%) at 3-month and 18.5% (95%CI, 15.2 to 21.8%) at 12-month follow-up.

### 3.1. Predictors of Penumbra Salvage

We report acute and subacute variables according to tertiles of PS in Supplemental Table S1.

In the multivariable ordinal logistic regression model of acute phase variables, BMI values, the presence of neglect, higher baseline penumbra and clot burden score were associated with increased tertiles of PS (Table 2). The presence of higher values of white blood cells (WBC), early ischemic changes and leukoaraiosis, PCA and/or ACA territory involved were associated with decreased tertiles of PS (Table 2). After inclusion of subacute variables, similar results were obtained (with a borderline association for WBC), in addition to recanalization status and occurrence of parenchymal hematoma (Table 2). In this model, recanalized patients had an increased PS compared with patients with no occlusion at admission and patients with parenchymal hematoma had a decreased PS compared with those without, though the difference did not reach the significance level (Table 2). We summarize the independent predictors of PS with common odds ratios for higher tertiles in volume change of penumbra in Table 2 (acute and acute plus subacute models).

In secondary analyses using a multivariable multinomial logistic regression model (using middle tertile of PS as reference category), the same independent acute predictors of PS were found except for WBC. In addition, compared with the middle tertile, the highest tertile of PS was associated with acute treatment and the lowest with presence of old infarct (Supplemental Figure S1).

### 3.2. Predictors of Infarct Growth

We report acute and subacute variables according to tertiles of IG in Supplemental Table S2.

In the multivariable ordinal logistic regression model of acute phase variables, current smoking, higher blood glucose values, the presence of early ischemic changes, leukoaraiosis, old infarct, hyperdense MCA, additional involvement of PCA and/or ACA territory and extracranial stenosis ≥ 50% or occlusion were associated with increased infarct core tertiles (Table 3). Baseline infarct core and higher clot burden score were associated with decreased infarct core tertiles.

When adding subacute variables, we obtained the same independent predictors of IG in multivariable analysis. In this model, recanalized patients had a decreased IG compared with patients with no occlusion at admission and patients with parenchyma hematoma had an increased IG compared with those without. The independent predictors of IG with common odds ratios for higher tertiles in volume change of infarct core are summarized in Table 3 (acute and acute plus subacute models).

We found comparable results in multivariable multinomial regression analysis (Supplemental Figure S2). In this additional analysis, the lowest and the highest tertile of IG were also associated with female sex.

### 3.3. Impact of Penumbra Salvage and Infarct Growth on Clinical Outcome

Higher values of PS were significantly associated with better outcomes over the entire mRS distribution (shift analysis) at 3 months. Per one-point mRS decrease (i.e., better outcome), using the lowest tertile of PS as reference, the fully adjusted ORs (95%CI) were 1.48 (0.93 to 2.34) for the middle tertile and 2.89 (1.65 to 5.06) for the upper tertile. We found similar associations for excellent and favourable outcomes and for 90-day all-cause mortality rate (Table 4).

Regarding IG, an inverse association was found with 90-day mRS shift analysis. Using the lowest tertile of IG as reference, the fully adjusted ORs (95%CI) for one-point mRS decrease were 0.61 (0.38 to 0.96) for the middle tertile and 0.27 (0.17 to 0.42) for the upper tertile. Similar associations were found for excellent and favourable outcomes and for 90-day all-cause mortality rate (Table 5).

These results were confirmed in the 12-month analyses for mRS shift, functional outcome and mortality (Supplemental Tables S3 and S4).

## 4. Discussion

In this large cohort of acute ischemic stroke patients, we identified multiple clinical, biological and radiological factors determining PS and IG, beyond recanalization. However, some factors affected only PS, while others affected IG and several others seemed to be implicated in both.

### 4.1. Factors Associated with Penumbra Salvage Only

Increased PS was associated with higher BMI and the presence of hemineglect, whereas a higher admission WBC count correlated with reduced PS. Among radiological variables, a larger baseline penumbra correlated strongly with increased PS.

Additionally known as the “obesity paradox”, previous studies have suggested that overweight patients with stroke or TIA have better survival and better functional status than patients with normal BMI [35]. Though our findings may partially elucidate this association, the mechanism explaining increased PS in overweight patients remains to be established.

We detected a positive association of neglect with PS that could contribute to the current debate on its anatomical network. Neglect appears to be anatomically related to large distributed networks of distinct and far-spread cortical regions [36]. The positive association of neglect with PS can therefore be explained by the initial disconnection of these networks from large volumes of ischemia that may have a high potential of reversibility.

The association of a higher admission WBC count with reduced PS may be related to the release of inflammatory mediators into the ischemic region. This mechanism has previously been shown to contribute to neuronal cell death, thereby exacerbating brain injury [37] and may constitute a target for future stroke therapies.

Finally, the initial penumbra burden was also associated with increased PS, despite baseline adjustment for this variable. A larger volume of tissue at risk may represent more tissue that is liable to recover after acute treatment. Similar findings have been reported by Cho et al. [38] and are likely explained by the approximatively 50% of (spontaneous or therapeutic) recanalization in our cohort, leading to a substantial extent of PS in these patients [11,18].

### 4.2. Factors Associated with Infarct Growth Only

A lesser IG was associated with non-smoking and a lower admission blood glucose. Among the radiological variables, the presence of a larger baseline infarct core correlated with reduced IG whereas the presence of extracranial arterial stenosis or occlusion was associated with increased IG. Finally, the intracerebral haemorrhage was associated with increased IG among subacute variables.

Several studies have previously shown an association between smoking and good clinical outcomes in stroke patients treated with intravenous thrombolysis, known as the smoking paradox [39]. However, Kurmann et al. have suggested that this apparent paradox is probably related to differences in the baseline characteristics of the patients, with no real clinical benefit [40]. In fact, as well as being a risk factor for stroke, smoking is also involved in dysfunction of blood brain barrier permeability and ion transporters leading to increased ischemic brain injury [41]. Consequently, smoking may play a crucial role in determining the infarct growth in such patients.

In our study, hyperglycaemia showed a positive association with lesion growth and final infarct size. This is consistent with the well-established negative impact of hyperglycaemia on clinical outcomes in acute ischemic stroke [42,43].

As previously described, baseline core volumes measured by CT or MRI were strong predictors of final lesion [12,44], independently of recanalization and of the initial penumbra volume. Likewise, several studies have found an association between large baseline ischemic core in acute ischemic stroke and high risk of poor outcomes after revascularization treatment [45,46]. This is why some patients develop a large infarct faster, with a large core volume on baseline imaging with less viable tissue, explaining the lesser IG at 24 h. Our findings could further explain the different evolutions of lesion volume during the first 24 h to target better responders to acute therapies.

In our study, we found higher IG in patients with extracranial arterial stenosis or occlusion. This result may be related to poorer collateral circulation supplying the ischemic region [47]. Indeed, in patients with stroke from large artery atherosclerosis, arterial stiffness may contribute to the collateral failure, losing the beneficial effects of acute treatments [48]. We could also suggest that the presence of extracranial arterial pathology was a stronger independent predictor of IG than collateral status by itself. Furthermore, patients with tandem lesions show poorer recanalization, independently of performing acute treatment [31,49], as well as poorer clinical outcome [50,51,52]. Whether very aggressive acute recanalization strategies for such patients could prevent this increased risk of IG and poorer outcome needs further assessment.

The association between haemorrhagic transformation and initial infarct size and severity [53,54,55] is well established. We observed an additional, independent association between haemorrhagic transformation (in the form of parenchymal hematoma) and increased IG which may be explained by factors determining haemorrhage in patients with large infarct core and severe hypoperfusion, i.e., early endothelial dysfunction, breakdown of the blood–brain barrier, and oxidative stress [56,57,58]. On the other hand, haemorrhagic transformation could also promote IG through cytotoxic effects of the extravasated blood [59,60]. Nevertheless, our findings suggest that preventing IG may improve outcome by averting parenchymal hematoma.

### 4.3. Factors Associated with Both Penumbra Salvage and Infarct Growth

In our study, several radiological factors were associated with both PS and IG. Among acute variables, a higher clot burden score was associated with increased PS and reduced IG. The presence of multiple vascular territory involvement, early ischemic changes, leukoaraiosis and old infarct correlated with reduced PS and increased IG. Among subacute variables, recanalization was associated with increased PS and reduced IG.

The demonstrated association between the clot burden and PS/IG is consistent with the previously described correlation between proximal and longer clots and poorer outcomes [61]. In these cases of lower CBS, lower recanalization rates have been reported, independently of acute treatments such as thrombolysis and thrombectomy [62]. With increasing CBS (i.e., less thrombus burden), patients were significantly more likely to have a better functional outcome, consistent with our findings.

The involvement of multiple vascular territories (adjacent territories to the MCA) leading to reduced PS and increased IG is in line with previous observations [63]. Indeed, Kaesmacher et al. have reported that multiple vascular occlusions are predictive of lower rates of successful reperfusion, leading to worse clinical outcomes—possibly to take into account treatment decisions [63].

A decreased attenuation on NCCT correlates with the DWI lesion and reduced CBV on MRI [64,65,66]. Thus, the presence of early ischemic changes probably corresponds to hypoperfusion and is likely to result in infarction, with previous studies reporting poor outcomes if they are anatomically extensive [67,68]. This is well supported by our observed association between reduced PS and increased IG.

The presence of leukoaraiosis and old infarct also negatively influenced PS (and positively IG), independently of initial lesion size and other factors, as previously described [69]. The presence of white matter lesions is a marker of chronic cerebrovascular injury and its burden may lead to a higher susceptibility of cerebral tissue to ischemia [70]. This could suggest a reduced capacity of penumbra regions to survive in these patients.

Unexpectedly, and unlike other studies [71], we did not find a significant association between good collaterals and IG/PS. While not a substitute for perfusion imaging, the collateral grade has been considered a surrogate of the cortical hypoperfusion [72] and the size of core infarct [73]. Nevertheless, we had already found in a previous analysis that, in MCA occlusions, better collaterals were not associated with higher penumbra volumes, suggesting a major role of collaterals in early tissue loss and their limited significance as a marker of salvageable tissue [74]. While collateral status is considered a key determinant of ischemic core and penumbra [75,76], and better collaterals seem to be associated with the greater benefit of IVT and EVT [77], recent data have questioned this assumption, suggesting that collateral status does not modify the association between successful recanalization and functional outcome [78].

Other probable factors could explain this finding. In our comprehensive multivariate analysis, we were able to properly detect stronger independent predictors of IG and PS beyond collaterals, such as baseline core and penumbra volumes, as well as brain frailty and widespread involvement, clot burden score and recanalization.

As is well known, recanalization correlates strongly with increased PS and with reduced IG [11,71,79]. This finding underlines the beneficial clinical effect of a complete recanalization, in particular when salvageable tissue is identified by neuroimaging [14,80].

Of note, delay from stroke onset to imaging within the first 24 h did not show association with subsequent PS and IG, confirming that prediction of tissue fate by acute imaging can be applied irrespectively of time delays [81]. In the era of late treatments, time-dependent factors that may influence a patient’s outcome are becoming less relevant. These findings strongly suggest that treatment decisions based on initial tissue viability may be associated with better patient outcomes than time-based selection.

### 4.4. Outcome

We showed a strong independent correlation of increased PS and reduced IG with multiple measures of clinical outcome at 3 months, such as functional outcome and mortality. Other authors have previously reported such associations, however we extended these observations to 12 months and included mortality rate [82,83]. This suggests that modifiable factors associated with PS and IG should be targeted by appropriate early management, such as rapid recanalization, treatment of tandem lesions, and elevated blood glucose, as they should improve the patient’s selection for more aggressive therapies.

Limitations of our analysis mainly relate to its retrospective design and single-centre data. Although technical limitations prevented inclusion of consecutive MCA patients in the series, the demographics and clinical profile of our patients (Table 1) are quite representative of MCA patients arriving at a comprehensive stroke centre. A power calculation was not performed; however, the 551 patients available for analysis should allow the detection of an important number of significant differences. The threshold model we used for core and penumbra volumes is a well-established model based on systematic evaluation of all PCT parameters [21]. Other threshold models have been developed [84], though the most accurate model remains to be established due to the limited availability of direct comparisons [85]. Furthermore, though CTP technology has evolved over the long observation period, we performed most exams after 11/2005 with homogeneous acquisition techniques and identical thresholds applied. Similarly, revascularisation techniques have changed over time and most of our data stem from the thrombolysis era. However, our substantial overall 24 h recanalization rate of about 50% allows for the meaningful analysis of this variable as a predictor of tissue fate.

## 5. Conclusions

In this comprehensive analysis, we have identified multiple clinical, biological and radiological factors that influence tissue fate in acute ischemic stroke. In particular, several radiological features predicted increased PS and reduced IG, such as brain frailty, increased vascular territory involvement and successful recanalization. Surprisingly, we did not find an association between the collateral status and IG/PS—other variables seemed to be more important. Thus, patients across all collateral grades might still benefit from reperfusion therapies depending on other factors. Finally, the delay from stroke onset to imaging within the first 24 h did not affect the PS and IG, while initial core and penumbra volumes were strongly associated with increased PS and reduced IG, confirming the “penumbra is brain” paradigm [86]. The magnitude of the independent correlation of PS and IG with functional outcome and mortality was powerful. We could suggest that focusing on the individual patient assessment, especially identifying such radiological predictors of PS/IG, may allow for the improved targeting of patients for later or more appropriate therapeutic approaches.

## Figures and Tables

**Table 1:** Patient characteristics, clinical and radiological findings and outcomes of overall study population (N = 551).

	N	Values
Acute Variables		
*Demographics*		
Age (years)	551	66.3 (14.7)
Males	551	280 (50.8)
*Medical history*		
Hypertension	551	340 (61.7)
Dyslipidemia	549	360 (65.6)
Diabetes	550	82 (14.9)
Current smoking	547	148 (27.1)
Previous stroke or TIA	551	103 (18.7)
Atrial fibrillation	550	185 (33.6)
Treatment before stroke	550	185 (33.6)
Lipid-lowering drug	551	122 (22.1)
Anti-hypertensive drug	547	282 (51.6)
Anti-diabetic drug	551	48 (8.7)
Antiplatelet	551	171 (31.0)
Anticoagulant	551	52 (9.4)
Pre-stroke mRS	551	155 (28.1)
*Clinical measurements*		
Body temperature (°C)	537	36.3 (0.7)
Systolic Blood Pressure (mmHg)	549	155 (28)
BMI (kg/m^2^)	398	25.6 (4.6)
*Biological data*		
Blood glucose (mmol/L)	540	6.6 (5.8 to 7.8)
Total cholesterol (mmol/L)	496	5.38 (1.75)
WBC count (G/L)	540	8.0 (6.5 to 10.2)
Hemoglobin (g/L)	540	138 (16)
Creatinine (µm/L)	543	89 (24)
*New neurological deficit*		
Admission NIHSS	551	13.3 (7.1)
Visual field defects	546	320 (58.6)
Eye deviation	546	231 (42.3)
Aphasia	547	270 (49.4)
Neglect	543	244 (44.9)
Vigilance impairment	539	75 (13.9)
Dysarthria	547	340 (62.2)
Paresis	548	506 (92.3)
Sensory loss	547	411 (75.1)
Simultaneous PCA and/or ACA involvement	551	44 (8.0)
Onset-to-door time (minutes)	550	131 (72 to 301)
Onset-to-CT time (minutes)	546	170 (102 to 385)
*Neuroimaging data*		
Baseline penumbra, mm	551	62.6 (23.4 to 108.7)
Baseline infarct core, mm	551	28.9 (8.1 to 75.7)
Early ischemic changes	551	303 (55.0)
ASPECTS	551	8 (6 to 10)
Old infarct	551	126 (22.9)
Leukoaraiosis	551	98 (17.8)
Hyperdense MCA	550	448 (81.5)
Clot burden score	442	6 (4 to 9.0)
Cerebral stenosis ≥ 50% or occlusion	550	448 (81.5)
Extracranial stenosis ≥ 50% or occlusion	550	170 (30.9)
Intracranial stenosis ≥ 50% or occlusion	550	414 (75.3)
Good collaterals	551	352 (63.9)
*Reperfusion therapy*	551	273 (49.6)
IV thrombolysis	551	256 (46.5)
Endovascular treatment	551	17 (3.1)
**Subacute variables**		
*Clinical and biological measurements*		
Systolic blood pressure (mmHg)	525	137 (21)
Body temperature (°C)	508	37 (0.7)
Blood glucose (mmol/L)	432	5.6 (4.9 to 6.5)
*Neuroimaging data*		
Final infarct	551	24.7 (5.8 to 72.9)
Penumbra salvage	551	59.3 (15.9 to 117.5)
Infarct growth	551	−0.0 (−15.1 to 18.4)
Recanalization	463	
No occlusion		131 (28.3)
Recanalized		218 (47.1)
Non-recanalized		114 (24.6)
Parenchymal hemorrhage	460	45 (9.8)
*TOAST Mechanism*	548	
Large artery atherosclerosis		83 (15.2)
Cardioembolism		230 (42.0)
SVD and other determined etiology ^1^		123 (22.4)
Undetermined etiology ^2^		
**3-month outcome**		
mRS	547	2 (1 to 4)
c-mRS 0–1	547	180 (32.9)
c-mRS 0–2	547	289 (52.8)
All-cause mortality	547	76 (13.9)
**12-month outcome**		
mRS	541	2 (1 to 4)
c-mRS 0–1	541	206 (38.1)
c-mRS 0–2	541	297 (54.9)
All-cause mortality	541	100 (18.5)

Values are n (%), mean (SD) or median (IQR). c-mRS defined as the difference in follow-up mRs and pre-stroke mRs evaluation. Abbreviations: TIA = transient ischemic attack, ACA = anterior cerebral artery, ASPECTS = Alberta stroke program early compute tomography score, BMI = body mass index, c-mRS = corrected modified Rankin score, IQR = interquartile range, MCA = middle cerebral artery, mRS = modified Rankin score, c-mRS = corrected-mRS, NIHSS = National Institutes of Health Stroke Scale, PCA = posterior cerebral artery, SD = standard deviation, TOAST = Trial of Org 10,172 in Acute Stroke Treatment, WBC = white blood cells. ^1^ included small vessel disease, dissection and other rare causes. ^2^ included unknow aetiologies or multiple causes.

**Table 2:** Independent predictors of penumbra salvage (categorized into tertiles).

Predictors	Acute Model		Acute and Subacute Models	
	OR (95%CI) ^1^	*p* Value	OR (95%CI) ^1^	*p* Value
**Acute characteristics**				
BMI (per 1 SD increase)	1.33 (1.06 to 1.67)	0.011	1.31 (1.04 to 1.65)	0.020
WBC (per 1 log SD increase)	0.80 (0.65 to 0.99)	0.032	0.82 (0.66 to 1.01)	0.056
Neglect	1.81 (1.22 to 2.68)	0.003	1.66 (1.11 to 2.49)	0.013
Baseline penumbra (per 1 log SD increase)	18.75 (12.48 to 28.17)	<0.001	18.05 (11.90 to 27.36)	<0.001
PCA and/or ACA involved	0.32 (0.15 to 0.68)	0.003	0.36 (0.17 to 0.77)	0.008
Early ischemic changes	0.55 (0.36 to 0.84)	0.005	0.56 (0.36 to 0.85)	0.006
Clot burden score (per 1 point increase)	1.09 (1.01 to 1.17)	0.016	1.13 (1.04 to 1.22)	0.003
Leukoaraiosis	0.44 (0.26 to 0.74)	0.001	0.46 (0.27 to 0.77)	0.003
**Subacute characteristics**				
Recanalization	Not entered	-		0.006 ^2^
No occlusion			1.00 (reference)	-
Recanalized			2.34 (1.27 to 4.29)	0.006
Non-recanalized			1.28 (0.64 to 2.56)	0.48
Parenchymal hemorrhage at 24 h	Not entered	-	0.56 (0.29 to 1.07)	0.078

^1^ Common odds ratio for higher tertiles in volume change of penumbra; ^2^ *p*-value for overall effect. Model 1 calculated after handling missing data by multiple imputation (m = 10) using a backward-stepwise ordinal logistic model including all univariate acute predictors at *p* < 0.10 (except admission NIHSS score since individual components were candidates). Model 2 calculated after handling missing data by multiple imputation (m = 10) using a backward-stepwise ordinal logistic model including all univariate acute and subacute predictors at *p* < 0.10 (except admission NIHSS score, as individual components were candidates). Abbreviation: ACA = anterior cerebral artery, BMI = body mass index, NIHSS = National Institutes of Health Stroke Scale, PCA = posterior cerebral artery, SD = standard deviation, WBC = white blood cells.

**Table 3:** Independent predictors of infarct growth (categorized into tertiles).

Predictors	Acute Model		Acute and Subacute Models	
	OR (95%CI) ^1^	*p* Value	OR (95%CI) ^1^	*p* Value
**Acute characteristics**				
Current smoking	1.56 (1.06 to 2.27)	0.022	1.54 (1.05 to 2.25)	0.027
Blood glucose (per 1 log SD increase)	1.35 (1.13 to 1.61)	<0.001	1.33 (1.11 to 1.59)	0.002
Baseline infarct core (per 1 log SD increase)	0.41 (0.32 to 0.51)	<0.001	0.44 (0.34 to 0.55)	<0.001
PCA and/or ACA involved	6.32 (3.10 to 12.88)	<0.001	5.76 (2.75 to 12.05)	<0.001
Early ischemic changes	2.26 (1.56 to 3.27)	<0.001	2.27 (1.55 to 3.31)	<0.001
Hyperdense MCA	1.80 (1.15 to 2.78)	0.009	1.91 (1.22 to 2.98)	0.004
Clot burden score (per 1 point increase)	0.91 (0.83 to 0.98)	0.015	0.87 (0.80 to 0.95)	0.001
Leukoaraiosis	2.04 (1.31 to 3.19)	0.002	2.05 (1.30 to 3.23)	0.002
Old infarct	1.55 (1.04 to 2.32)	0.031	1.66 (1.11 to 2.51)	0.015
Extracranial stenosis ≥ 50% or occlusion	1.64 (1.11 to 2.41)	0.012	1.55 (1.03 to 2.30)	0.032
Good collaterals	0.68 (0.45 to 1.03)	0.067	Not selected	-
**Subacute characteristics**				
Recanalization	Not entered	-		<0.001 ^2^
No occlusion			1.00 (reference)	-
Recanalized			0.50 (0.31 to 0.83)	0.006
Non-recanalized			1.11 (0.64 to 1.90)	0.72
Parenchymal hemorrhage at 24 h	Not entered	-	2.61 (1.39 to 4.87)	0.003

^1^ Common odds ratio for higher tertiles in change of infarct core; ^2^ *p*-value for overall effect. Model 1 calculated after handling missing data by multiple imputation (m = 10) using a backward-stepwise ordinal logistic model including all univariate acute predictors at *p* < 0.10 (except admission NIHSS score, as individual components were candidates). Model 2 calculated after handling missing data by multiple imputation (m = 10) using a backward-stepwise ordinal logistic model including all univariate acute and subacute predictors at *p* < 0.10 (except admission NIHSS score, as individual components were candidates). Abbreviation: ACA = anterior cerebral artery, MCA = middle cerebral artery, NIHSS = National Institutes of Health Stroke Scale, PCA = posterior cerebral artery, SD = standard deviation.

**Table 4:** Association of penumbra salvage tertiles with 3-month outcomes (overall degree of disability, excellent outcome, favourable outcome, and all-cause mortality).

	24 h Salvage of the Penumbra Volume (Median [Range], mL)			
	<33th Percentile	33–65th Percentiles	≥66th Percentiles	
	2.6 [−50.0 to 26.7]	59.3 [26.8 to 97.6]	139.8 [97.7 to 250.0]	
	(N = 183)	(N = 184)	(N = 184)	*p*-Value ^1^
**mRS (shift analysis)**				
Median (IQR)	2 (1–4)	3 (1–4)	2 (1–3)	
Baseline adjusted OR (95%CI) ^2^	1.00 (reference)	2.20 (1.43 to 3.36)	4.94 (2.96 to 8.25)	<0.0001
Fully adjusted OR (95%CI) ^3^	1.00 (reference)	1.48 (0.93 to 2.34)	2.89 (1.65 to 5.06)	0.0001
**Corrected mRS 0–1**				
Baseline adjusted rates, %	18.4	35.0	45.9	
Baseline adjusted OR (95%CI)	1.00 (reference)	2.39 (1.36 to 4.19)	3.76 (1.89 to 7.43)	<0.0001
Fully adjusted OR (95%CI) ^3^	1.00 (reference)	1.97 (1.00 to 3.87)	3.44 (1.46 to 8.11)	0.005
**Corrected mRS 0–2**				
Baseline adjusted rates, %	35.4	52.4	71.1	
Baseline adjusted OR (95%CI)	1.00 (reference)	2.01 (1.20 to 3.33)	4.48 (2.41 to 8.31)	<0.0001
Fully adjusted OR (95%CI) ^3^	1.00 (reference)	1.32 (0.71 to 2.46)	2.78 (1.30 to 5.93)	0.004
**All-cause mortality**				
Baseline adjusted rates, %	31.3	13.5	2.5	
Baseline adjusted OR (95%CI)	1.00 (reference)	0.34 (0.18 to 0.63)	0.06 (0.02 to 0.15)	<0.0001
Fully adjusted OR (95%CI) ^3^	1.00 (reference)	0.47 (0.22 to 0.98)	0.10 (0.03 to 0.29)	<0.0001

^1^ *p*-values calculated by including the tertiles of volume change in penumbra as an ordinal variable into logistic regression models. ^2^ Common OR for 1-point improvement in mRs. ^3^ Adjusted for predictors of penumbra salvage (baseline penumbra, leukoaraiosis, PCA and/or ACA involved, neglect, early ischemic changes, clot burden score, BMI, WBC, recanalization status, and parenchymal haemorrhage at 24 h) (calculated after handling missing data by multiple imputation procedure (m = 10 imputations)). Abbreviation: ACA = anterior cerebral artery, BMI = body mass index, CI = confidence interval, c-mRS = corrected modified Rankin score, mRS = modified ranking score, OR = odds ratio, PCA = posterior cerebral artery, WBC = white blood cells.

**Table 5:** Association of infarct growth tertiles with 3-month outcomes (overall degree of disability, excellent outcome, favourable outcome, and all-cause mortality).

	24 h Increase of the Infarct Core Median [Range], mL)			
	<33th Percentile	33–65th Percentiles	≥66th Percentiles	
	−25.7 [−50.0 to −7.3]	−0.0 [−7.2 to 8.4]	35.0 [18.4 to 250.0]	
	(N = 183)	(N = 184)	(N = 184)	*p*-Value ^1^
**mRS (shift analysis)**				
median (IQR)	2 (1–3)	2 (1–3)	4 (2–6)	
Baseline adjusted OR (95%CI) ^2^	1.00 (reference)	0.45 (0.29 to 0.69)	0.14 (0.09 to 0.21)	<0.0001
Fully adjusted OR (95%CI) ^3^	1.00 (reference)	0.61 (0.38 to 0.96)	0.27 (0.17 to 0.42)	<0.0001
**Corrected mRS 0–1**				
Baseline adjusted rates, %	51.6	31.7	10.3	
Baseline adjusted OR (95%CI)	1.00 (reference)	0.44 (0.25 to 0.75)	0.11 (0.05 to 0.20)	<0.0001
Fully adjusted OR (95%CI) ^3^	1.00 (reference)	0.66 (0.35 to 1.23)	0.22 (0.11 to 0.44)	<0.0001
**Corrected mRS 0–2**				
Baseline adjusted rates, %	72.7	56.6	28.7	
Baseline adjusted OR (95%CI)	1.00 (reference)	0.49 (0.29 to 0.82)	0.15 (0.09 to 0.25)	<0.0001
Fully adjusted OR (95%CI) ^3^	1.00 (reference)	0.69 (0.38 to 1.24)	0.29 (0.16 to 0.53)	<0.0001
**All-cause mortality**				
Baseline adjusted rates, %	3.9	9.0	21.9	
Baseline adjusted OR (95%CI)	1.00 (reference)	2.43 (1.03 to 5.70)	6.87 (3.47 to 13.61)	<0.0001
Fully adjusted OR (95%CI) ^3^	1.00 (reference)	2.37 (0.92 to 6.10)	4.29 (1.90 to 9.63)	0.0003

^1^ *p*-values calculated by including the tertiles of volume change in penumbra as an ordinal variable into logistic regression models. ^2^ Common OR for 1-point improvement in mRs. ^3^ Adjusted for predictors of penumbra salvage (baseline infarct core, early ischemic changes, PCA and/or ACA involved, acute blood glucose, leukoaraiosis, hyperdense MCA, extracranial stenosis ≥ 50% or occlusion, clot burden score, current smoking, old infarct, recanalization status, and parenchymal haemorrhage at 24 h) (calculated after handling missing data by multiple imputation procedure (m = 10 imputations)). Abbreviation: ACA = anterior cerebral artery, CI = confidence interval, c-mRs = corrected modified Rankin score, MCA = middle cerebral artery, mRs = modified Rankin score, OR = odds ratio, PCA = posterior cerebral artery.

## Data Availability

The data that support the findings of this study are available from the corresponding author, upon reasonable request.

## Supplementary Materials

The following supporting information can be downloaded at: https://www.mdpi.com/article/10.3390/jcm12144561/s1, Table S1: Univariate association of penumbra salvage with acute and subacute characteristics; Table S2: Univariate association of infarct growth with acute and subacute characteristics; Table S3: Association of Penumbra Salvage with 12-month outcomes (overall degree of disability, excellent outcome, favorable outcome, and all-cause mortality); Table S4: Association of Infarct Growth with 12-month outcomes (overall degree of disability, excellent outcome, favorable outcome, and all-cause mortality); Figure S1: Results of multivariate analysis assessing of penumbra salvage categorized into 3-level categorical variable according to tertiles of 24-hours volume change; Figure S2: Results of multivariate analysis assessing predictors of infarct growth categorized into 3-level categorical variable according to tertiles of change in infarct core.

## References

[1] Ackerman R.H., Correia J.A., Alpert N.M., Baron J.C., Gouliamos A., Grotta J.C., Brownell G.L., Taveras J.M. (1981). Positron imaging in ischemic stroke disease using compounds labeled with oxygen 15. Initial results of clinicophysiologic correlations. Arch. Neurol..

[2] Baron J.C., Bousser M.G., Comar D., Soussaline F., Castaigne P. (1981). Noninvasive tomographic study of cerebral blood flow and oxygen metabolism in vivo. Potentials, limitations, and clinical applications in cerebral ischemic disorders. Eur. Neurol..

[3] Lenzi G.L., Frackowiak R.S., Jones T. (1982). Cerebral oxygen metabolism and blood flow in human cerebral ischemic infarction. J. Cereb. Blood Flow Metab..

[4] Powers W.J., Grubb R.L., Darriet D., Raichle M.E. (1985). Cerebral blood flow and cerebral metabolic rate of oxygen requirements for cerebral function and viability in humans. J. Cereb. Blood Flow Metab..

[5] Rosner G., Graf R., Kataoka K., Heiss W.D. (1986). Selective functional vulnerability of cortical neurons following transient MCA-occlusion in the cat. Stroke.

[6] Mabuchi T., Lucero J., Feng A., Koziol J.A., del Zoppo G.J. (2005). Focal cerebral ischemia preferentially affects neurons distant from their neighboring microvessels. J. Cereb. Blood Flow Metab..

[7] del Zoppo G.J., Sharp F.R., Heiss W.D., Albers G.W. (2011). Heterogeneity in the penumbra. J. Cereb. Blood Flow Metab..

[8] Olivot J.M., Mlynash M., Thijs V.N., Purushotham A., Kemp S., Lansberg M.G., Wechsler L., Gold G.E., Bammer R., Marks M.P. (2009). Geography, structure, and evolution of diffusion and perfusion lesions in Diffusion and perfusion imaging Evaluation For Understanding Stroke Evolution (DEFUSE). Stroke.

[9] Albers G.W., Thijs V.N., Wechsler L., Kemp S., Schlaug G., Skalabrin E., Bammer R., Kakuda W., Lansberg M.G., Shuaib A. (2006). Magnetic resonance imaging profiles predict clinical response to early reperfusion: The diffusion and perfusion imaging evaluation for understanding stroke evolution (DEFUSE) study. Ann. Neurol..

[10] Lansberg M.G., Lee J., Christensen S., Straka M., De Silva D.A., Mlynash M., Campbell B.C., Bammer R., Olivot J.M., Desmond P. (2011). RAPID automated patient selection for reperfusion therapy: A pooled analysis of the Echoplanar Imaging Thrombolytic Evaluation Trial (EPITHET) and the Diffusion and Perfusion Imaging Evaluation for Understanding Stroke Evolution (DEFUSE) Study. Stroke.

[11] Lansberg M.G., Straka M., Kemp S., Mlynash M., Wechsler L.R., Jovin T.G., Wilder M.J., Lutsep H.L., Czartoski T.J., Bernstein R.A. (2012). MRI profile and response to endovascular reperfusion after stroke (DEFUSE 2): A prospective cohort study. Lancet Neurol..

[12] Man S., Aoki J., Hussain M.S., Wisco D., Tateishi Y., Toth G., Hui F.K., Uchino K. (2015). Predictors of infarct growth after endovascular therapy for acute ischemic stroke. J. Stroke Cerebrovasc. Dis..

[13] Nogueira R.G., Jadhav A.P., Haussen D.C., Bonafe A., Budzik R.F., Bhuva P., Yavagal D.R., Ribo M., Cognard C., Hanel R.A. (2018). Thrombectomy 6 to 24 Hours after Stroke with a Mismatch between Deficit and Infarct. N. Engl. J. Med..

[14] Albers G.W., Marks M.P., Kemp S., Christensen S., Tsai J.P., Ortega-Gutierrez S., McTaggart R.A., Torbey M.T., Kim-Tenser M., Leslie-Mazwi T. (2018). Thrombectomy for Stroke at 6 to 16 Hours with Selection by Perfusion Imaging. N. Engl. J. Med..

[15] Thomalla G., Boutitie F., Ma H., Koga M., Ringleb P., Schwamm L.H., Wu O., Bendszus M., Bladin C.F., Campbell B.C.V. (2020). Intravenous alteplase for stroke with unknown time of onset guided by advanced imaging: Systematic review and meta-analysis of individual patient data. Lancet.

[16] Schwarz G., Agostoni E.C., Saliou G., Hajdu S.D., Salerno A., Dunet V., Michel P., Strambo D. (2023). Perfusion Imaging Mismatch Profiles in the Early Thrombectomy Window: A Single-Center Analysis. Stroke.

[17] Albers G.W. (2018). Late Window Paradox. Stroke.

[18] Zhu G., Michel P., Aghaebrahim A., Patrie J.T., Xin W., Eskandari A., Zhang W., Wintermark M. (2013). Prediction of recanalization trumps prediction of tissue fate: The penumbra: A dual-edged sword. Stroke.

[19] Vagal A., Aviv R., Sucharew H., Reddy M., Hou Q., Michel P., Jovin T., Tomsick T., Wintermark M., Khatri P. (2018). Collateral Clock Is More Important Than Time Clock for Tissue Fate. Stroke.

[20] Michel P., Odier C., Rutgers M., Reichhart M., Maeder P., Meuli R., Wintermark M., Maghraoui A., Faouzi M., Croquelois A. (2010). The Acute STroke Registry and Analysis of Lausanne (ASTRAL): Design and baseline analysis of an ischemic stroke registry including acute multimodal imaging. Stroke.

[21] Wintermark M., Flanders A.E., Velthuis B., Meuli R., van Leeuwen M., Goldsher D., Pineda C., Serena J., van der Schaaf I., Waaijer A. (2006). Perfusion-CT assessment of infarct core and penumbra: Receiver operating characteristic curve analysis in 130 patients suspected of acute hemispheric stroke. Stroke.

[22] Von Elm E., Altman D.G., Egger M., Pocock S.J., Gotzsche P.C., Vandenbroucke J.P., Initiative S. (2007). The Strengthening the Reporting of Observational Studies in Epidemiology (STROBE) statement: Guidelines for reporting observational studies. Epidemiology.

[23] Adams H.P., Bendixen B.H., Kappelle L.J., Biller J., Love B.B., Gordon D.L., Marsh E.E. (1993). Classification of subtype of acute ischemic stroke. Definitions for use in a multicenter clinical trial. TOAST. Trial of Org 10172 in Acute Stroke Treatment. Stroke.

[24] European Stroke Organisation Executive C., Committee E.S.O.W. (2008). Guidelines for management of ischaemic stroke and transient ischaemic attack 2008. Cerebrovasc. Dis..

[25] Michel P., Engelter S., Arnold M., Hungerbühler H.J., Nedeltchev K., Georgiadis D., Müller F., Bönig L., Müller M., Barth A. (2009). Thrombolyse de l’attaque cérébrale ischémique: Recommandations actualisées. Swiss Med. Forum.

[26] Blennow K., Wallin A., Uhlemann C., Gottfries C.G. (1991). White-matter lesions on CT in Alzheimer patients: Relation to clinical symptomatology and vascular factors. Acta Neurol. Scand..

[27] Puetz V., Dzialowski I., Hill M.D., Demchuk A.M. (2009). The Alberta Stroke Program Early CT Score in clinical practice: What have we learned?. Int. J. Stroke.

[28] Puetz V., Dzialowski I., Hill M.D., Subramaniam S., Sylaja P.N., Krol A., O’Reilly C., Hudon M.E., Hu W.Y., Coutts S.B. (2008). Intracranial thrombus extent predicts clinical outcome, final infarct size and hemorrhagic transformation in ischemic stroke: The clot burden score. Int. J. Stroke.

[29] Tan J.C., Dillon W.P., Liu S., Adler F., Smith W.S., Wintermark M. (2007). Systematic comparison of perfusion-CT and CT-angiography in acute stroke patients. Ann. Neurol..

[30] Hacke W., Kaste M., Fieschi C., von Kummer R., Davalos A., Meier D., Larrue, Bluhmki E., Davis S., Donnan G. (1998). Randomised double-blind placebo-controlled trial of thrombolytic therapy with intravenous alteplase in acute ischaemic stroke (ECASS II). Second European-Australasian Acute Stroke Study Investigators. Lancet.

[31] Vanacker P., Lambrou D., Eskandari A., Maeder P., Meuli R., Ntaios G., Michel P. (2014). Improving prediction of recanalization in acute large-vessel occlusive stroke. J. Thromb. Haemost..

[32] Allison P.D. (1998). Multiple Regression: A Primer.

[33] Van Buuren S., Oudshoorn C.G. (2011). Multivariate Imputation by Chained Equations in R. Journal of Statistical Software.

[34] Rubin D. (1987). Multivariate Imputation for Nonresponse in Surveys.

[35] Doehner W., Schenkel J., Anker S.D., Springer J., Audebert H.J. (2013). Overweight and obesity are associated with improved survival, functional outcome, and stroke recurrence after acute stroke or transient ischaemic attack: Observations from the TEMPiS trial. Eur. Heart J..

[36] Bartolomeo P., Thiebaut de Schotten M., Doricchi F. (2007). Left unilateral neglect as a disconnection syndrome. Cereb. Cortex.

[37] Kim J.Y., Park J., Chang J.Y., Kim S.H., Lee J.E. (2016). Inflammation after Ischemic Stroke: The Role of Leukocytes and Glial Cells. Exp. Neurobiol..

[38] Cho T.H., Nighoghossian N., Mikkelsen I.K., Derex L., Hermier M., Pedraza S., Fiehler J., Ostergaard L., Berthezene Y., Baron J.C. (2015). Reperfusion within 6 hours outperforms recanalization in predicting penumbra salvage, lesion growth, final infarct, and clinical outcome. Stroke.

[39] Meseguer E., Labreuche J., Gonzalez-Valcarcel J., Sirimarco G., Guidoux C., Cabrejo L., Lavallee P.C., Klein I.F., Amarenco P., Mazighi M. (2014). The smoking paradox: Impact of smoking on recanalization in the setting of intra-arterial thrombolysis. Cerebrovasc. Dis. Extra.

[40] Kurmann R., Engelter S.T., Michel P., Luft A.R., Wegener S., Branscheidt M., Eskioglou E., Sirimarco G., Lyrer P.A., Gensicke H. (2018). Impact of Smoking on Clinical Outcome and Recanalization After Intravenous Thrombolysis for Stroke: Multicenter Cohort Study. Stroke.

[41] Sifat A.E., Vaidya B., Villalba H., Albekairi T.H., Abbruscato T.J. (2018). Neurovascular unit transport responses to ischemia and common coexisting conditions: Smoking and diabetes. Am. J. Physiol. Cell Physiol..

[42] Ntaios G., Egli M., Faouzi M., Michel P. (2010). J-shaped association between serum glucose and functional outcome in acute ischemic stroke. Stroke.

[43] Bruno A., Levine S.R., Frankel M.R., Brott T.G., Lin Y., Tilley B.C., Lyden P.D., Broderick J.P., Kwiatkowski T.G., Fineberg S.E. (2002). Admission glucose level and clinical outcomes in the NINDS rt-PA Stroke Trial. Neurology.

[44] Ribo M., Tomasello A., Lemus M., Rubiera M., Vert C., Flores A., Coscojuela P., Pagola J., Rodriguez-Luna D., Bonet S. (2015). Maximal Admission Core Lesion Compatible with Favorable Outcome in Acute Stroke Patients Undergoing Endovascular Procedures. Stroke.

[45] Olivot J.M., Mosimann P.J., Labreuche J., Inoue M., Meseguer E., Desilles J.P., Rouchaud A., Klein I.F., Straka M., Bammer R. (2013). Impact of diffusion-weighted imaging lesion volume on the success of endovascular reperfusion therapy. Stroke.

[46] Wheeler H.M., Mlynash M., Inoue M., Tipirnini A., Liggins J., Bammer R., Lansberg M.G., Kemp S., Zaharchuk G., Straka M. (2015). The growth rate of early DWI lesions is highly variable and associated with penumbral salvage and clinical outcomes following endovascular reperfusion. Int. J. Stroke.

[47] Kluytmans M., van der Grond J., van Everdingen K.J., Klijn C.J., Kappelle L.J., Viergever M.A. (1999). Cerebral hemodynamics in relation to patterns of collateral flow. Stroke.

[48] Acampa M., Romano D.G., Lazzerini P.E., Leonini S., Guideri F., Tassi R., Casseri T., Bracco S., Martini G. (2018). Increased Arterial Stiffness is Associated with Poor Collaterals in Acute Ischemic Stroke from Large Vessel Occlusion. Curr. Neurovasc. Res..

[49] Vanacker P., Heldner M.R., Seiffge D., Mueller H., Eskandari A., Traenka C., Ntaios G., Mosimann P.J., Sztajzel R., Mendes Pereira V. (2015). ASTRAL-R score predicts non-recanalisation after intravenous thrombolysis in acute ischaemic stroke. Thromb. Haemost..

[50] De Silva D.A., Brekenfeld C., Ebinger M., Christensen S., Barber P.A., Butcher K.S., Levi C.R., Parsons M.W., Bladin C.F., Donnan G.A. (2010). The benefits of intravenous thrombolysis relate to the site of baseline arterial occlusion in the Echoplanar Imaging Thrombolytic Evaluation Trial (EPITHET). Stroke.

[51] Mokin M., Kass-Hout T., Kass-Hout O., Dumont T.M., Kan P., Snyder K.V., Hopkins L.N., Siddiqui A.H., Levy E.I. (2012). Intravenous thrombolysis and endovascular therapy for acute ischemic stroke with internal carotid artery occlusion: A systematic review of clinical outcomes. Stroke.

[52] Kappelhof M., Marquering H.A., Berkhemer O.A., Majoie C.B. (2015). Intra-arterial treatment of patients with acute ischemic stroke and internal carotid artery occlusion: A literature review. J. Neurointerv. Surg..

[53] Jain A.R., Jain M., Kanthala A.R., Damania D., Stead L.G., Wang H.Z., Jahromi B.S. (2013). Association of CT perfusion parameters with hemorrhagic transformation in acute ischemic stroke. AJNR. Am. J. Neuroradiol..

[54] Yassi N., Parsons M.W., Christensen S., Sharma G., Bivard A., Donnan G.A., Levi C.R., Desmond P.M., Davis S.M., Campbell B.C. (2013). Prediction of poststroke hemorrhagic transformation using computed tomography perfusion. Stroke.

[55] Tsetsou S., Amiguet M., Eskandari A., Meuli R., Maeder P., Jiang B., Wintermark M., Michel P. (2017). Severe cerebral hypovolemia on perfusion CT and lower body weight are associated with parenchymal haemorrhage after thrombolysis. Neuroradiology.

[56] del Zoppo G.J., von Kummer R., Hamann G.F. (1998). Ischaemic damage of brain microvessels: Inherent risks for thrombolytic treatment in stroke. J. Neurol. Neurosurg. Psychiatry.

[57] Hamann G.F., del Zoppo G.J., von Kummer R. (1999). Hemorrhagic transformation of cerebral infarction--possible mechanisms. Thromb. Haemost..

[58] Lee S.J., Lee K.H., Na D.G., Byun H.S., Kim Y.B., Shon Y.M., Cho S.J., Lee J., Chung C.S., Hong S.C. (2004). Multiphasic helical computed tomography predicts subsequent development of severe brain edema in acute ischemic stroke. Arch. Neurol..

[59] Aronowski J., Zhao X. (2011). Molecular pathophysiology of cerebral hemorrhage: Secondary brain injury. Stroke.

[60] Zheng H., Chen C., Zhang J., Hu Z. (2016). Mechanism and Therapy of Brain Edema after Intracerebral Hemorrhage. Cerebrovasc. Dis..

[61] Legrand L., Naggara O., Turc G., Mellerio C., Roca P., Calvet D., Labeyrie M.A., Baron J.C., Mas J.L., Meder J.F. (2013). Clot burden score on admission T2*-MRI predicts recanalization in acute stroke. Stroke.

[62] Heo J.H., Kim K., Yoo J., Kim Y.D., Nam H.S., Kim E.Y. (2017). Computed Tomography-Based Thrombus Imaging for the Prediction of Recanalization after Reperfusion Therapy in Stroke. J. Stroke.

[63] Kaesmacher J., Mosimann P.J., Giarrusso M., El-Koussy M., Zibold F., Piechowiak E., Dobrocky T., Meier R., Jung S., Bellwald S. (2018). Multivessel Occlusion in Patients Subjected to Thrombectomy: Prevalence, Associated Factors, and Clinical Implications. Stroke.

[64] Barber P.A., Darby D.G., Desmond P.M., Gerraty R.P., Yang Q., Li T., Jolley D., Donnan G.A., Tress B.M., Davis S.M. (1999). Identification of major ischemic change. Diffusion-weighted imaging versus computed tomography. Stroke.

[65] Kucinski T., Vaterlein O., Glauche V., Fiehler J., Klotz E., Eckert B., Koch C., Rother J., Zeumer H. (2002). Correlation of apparent diffusion coefficient and computed tomography density in acute ischemic stroke. Stroke.

[66] Sobesky J., von Kummer R., Frackowiak M., Zaro Weber O., Lehnhardt F.G., Dohmen C., Neveling M., Moller-Hartmann W., Jacobs A.H., Heiss W.D. (2006). Early ischemic edema on cerebral computed tomography: Its relation to diffusion changes and hypoperfusion within 6 h after human ischemic stroke. A comparison of CT, MRI and PET. Cerebrovasc. Dis..

[67] Barber P.A., Demchuk A.M., Zhang J., Buchan A.M. (2000). Validity and reliability of a quantitative computed tomography score in predicting outcome of hyperacute stroke before thrombolytic therapy. ASPECTS Study Group. Alberta Stroke Programme Early CT Score. Lancet.

[68] Dzialowski I., Hill M.D., Coutts S.B., Demchuk A.M., Kent D.M., Wunderlich O., von Kummer R. (2006). Extent of early ischemic changes on computed tomography (CT) before thrombolysis: Prognostic value of the Alberta Stroke Program Early CT Score in ECASS II. Stroke.

[69] Ay H., Arsava E.M., Rosand J., Furie K.L., Singhal A.B., Schaefer P.W., Wu O., Gonzalez R.G., Koroshetz W.J., Sorensen A.G. (2008). Severity of leukoaraiosis and susceptibility to infarct growth in acute stroke. Stroke.

[70] Rost N.S., Fitzpatrick K., Biffi A., Kanakis A., Devan W., Anderson C.D., Cortellini L., Furie K.L., Rosand J. (2010). White matter hyperintensity burden and susceptibility to cerebral ischemia. Stroke.

[71] Jung S., Gilgen M., Slotboom J., El-Koussy M., Zubler C., Kiefer C., Luedi R., Mono M.L., Heldner M.R., Weck A. (2013). Factors that determine penumbral tissue loss in acute ischaemic stroke. Brain.

[72] Seyman E., Shaim H., Shenhar-Tsarfaty S., Jonash-Kimchi T., Bornstein N.M., Hallevi H. (2016). The collateral circulation determines cortical infarct volume in anterior circulation ischemic stroke. BMC Neurol..

[73] Cheng-Ching E., Frontera J.A., Man S., Aoki J., Tateishi Y., Hui F.K., Wisco D., Ruggieri P., Hussain M.S., Uchino K. (2015). Degree of Collaterals and Not Time Is the Determining Factor of Core Infarct Volume within 6 Hours of Stroke Onset. Am. J. Neuroradiol..

[74] Nannoni S., Cereda C.W., Sirimarco G., Lambrou D., Strambo D., Eskandari A., Dunet V., Wintermark M., Michel P. (2019). Collaterals are a major determinant of the core but not the penumbra volume in acute ischemic stroke. Neuroradiology.

[75] Berkhemer O.A., Jansen I.G., Beumer D., Fransen P.S., van den Berg L.A., Yoo A.J., Lingsma H.F., Sprengers M.E., Jenniskens S.F., Lycklama A.N.G.J. (2016). Collateral Status on Baseline Computed Tomographic Angiography and Intra-Arterial Treatment Effect in Patients with Proximal Anterior Circulation Stroke. Stroke.

[76] Venema E., Roozenbeek B., Mulder M., Brown S., Majoie C., Steyerberg E.W., Demchuk A.M., Muir K.W., Davalos A., Mitchell P.J. (2021). Prediction of Outcome and Endovascular Treatment Benefit: Validation and Update of the MR PREDICTS Decision Tool. Stroke.

[77] Uniken Venema S.M., Dankbaar J.W., van der Lugt A., Dippel D.W.J., van der Worp H.B. (2022). Cerebral Collateral Circulation in the Era of Reperfusion Therapies for Acute Ischemic Stroke. Stroke.

[78] Uniken Venema S.M., Dankbaar J.W., Wolff L., van Es A., Sprengers M., van der Lugt A., Dippel D.W.J., van der Worp H.B., MR CLEAN Registry Investigators (2023). Collateral status and recanalization after endovascular treatment for acute ischemic stroke. J. Neurointerv. Surg..

[79] Lev M.H., Segal A.Z., Farkas J., Hossain S.T., Putman C., Hunter G.J., Budzik R., Harris G.J., Buonanno F.S., Ezzeddine M.A. (2001). Utility of perfusion-weighted CT imaging in acute middle cerebral artery stroke treated with intra-arterial thrombolysis: Prediction of final infarct volume and clinical outcome. Stroke.

[80] Campbell B.C., Mitchell P.J., Kleinig T.J., Dewey H.M., Churilov L., Yassi N., Yan B., Dowling R.J., Parsons M.W., Oxley T.J. (2015). Endovascular therapy for ischemic stroke with perfusion-imaging selection. N. Engl. J. Med..

[81] Qiao Y., Zhu G., Patrie J., Xin W., Michel P., Eskandari A., Jovin T., Wintermark M. (2014). Optimal perfusion computed tomographic thresholds for ischemic core and penumbra are not time dependent in the clinically relevant time window. Stroke.

[82] Beaulieu C., de Crespigny A., Tong D.C., Moseley M.E., Albers G.W., Marks M.P. (1999). Longitudinal magnetic resonance imaging study of perfusion and diffusion in stroke: Evolution of lesion volume and correlation with clinical outcome. Ann. Neurol..

[83] Kim S.M., Kwon S.U., Kim J.S., Kang D.W. (2014). Early infarct growth predicts long-term clinical outcome in ischemic stroke. J. Neurol. Sci..

[84] Lin L., Bivard A., Krishnamurthy V., Levi C.R., Parsons M.W. (2016). Whole-Brain CT Perfusion to Quantify Acute Ischemic Penumbra and Core. Radiology.

[85] Austein F., Riedel C., Kerby T., Meyne J., Binder A., Lindner T., Huhndorf M., Wodarg F., Jansen O. (2016). Comparison of Perfusion CT Software to Predict the Final Infarct Volume After Thrombectomy. Stroke.

[86] Michel P., Bogousslavsky J. (2005). Penumbra is brain: No excuse not to perfuse. Ann. Neurol..

