# Supplementary material for: Predicting Penumbra Salvage and Infarct Growth in Acute Ischemic Stroke: A Multifactor Survival Game
Source: J Clin Med. 2023 Jul 8;12(14):4561. doi: 10.3390/jcm12144561 (PMC10380847; doi:10.3390/jcm12144561)
Supplement: Supplementary file 1 [file jcm-12-04561-s001.zip › jcm-2467139-supplementary.pdf]

SUPPLEMENTAL TABLES

Table S1. Univariate association of penumbra salvage with acute and subacute characteristics.

	24-hour change in penumbra volume, ml				
	**<33th percentile**	**33-66th percentiles**	**≥ 66th percentiles**		
Median (range)	2.6 (-50.0 to 26.7)	59.3 (26.8 to 97.6)	139.8 (97.7 to 250.0)		
Number of patients	183	184	184	P-Value[1]	P-Value[2]
**Acute Variables**					
Demographics					
Age (yrs)	66.2 (15.7)	67.2 (14.9)	65.5 (13.4)	0.66	0.56
Males	92 (50.3)	91 (49.5)	97 (52.7)	0.083	0.25
Medical history					
Hypertension	114 (62.3)	119 (64.7)	107 (58.2)	0.33	0.21
Dyslipidemia	125 (69.1)	120 (65.2)	115 (62.5)	0.78	0.81
Diabetes	28 (15.3)	27 (14.7)	27 (14.8)	0.31	0.58
Current smoking	43 (23.8)	56 (30.6)	49 (26.8)	0.25	0.14
Previous stroke or TIA	40 (21.9)	32 (17.4)	31 (16.9)	0.89	0.97
Atrial fibrillation	54 (29.7)	59 (32.1)	72 (39.1)	0.58	0.53
Treatment before stroke					
Lipid-lowering drug	48 (26.2)	31 (16.9)	43 (23.4)	0.17	0.23
Anti-hypertensive drug	95 (52.5)	99 (54.1)	88 (48.1)	0.48	0.45
Anti-diabetic drug	16 (8.7)	15 (8.2)	17 (9.2)	0.18	0.40
Antiplatelet	61 (33.3)	51 (27.7)	59 (32.1)	0.99	0.45
Anticoagulant	15 (8.2)	21 (11.4)	16 (8.7)	0.86	0.64
Pre-stroke mRS>0	53 (29.0)	55 (29.9)	47 (25.5)	0.51	0.86
Clinical measurements					
Body temperature (°C)	36.3 (0.7)	36.4 (0.7)	36.2 (0.6)	0.71	0.22
Systolic Blood Pressure (mmHg)	158 (28)	156 (27)	151 (27)	0.48	0.63
BMI (kg/m^2)	25.2 (4.7)	25.6 (4.7)	26.1 (4.3)	0.008	0.043
Biological data					
Blood glucose (mmol/L)	6.6 (5.8-8.1)	6.6 (5.8-7.6)	6.5 (5.8-7.6)	0.29 [3]	0.64 [3]
Total cholesterol (mmol/L)	5.4 (1.3)	5.4 (1.3)	5.2 (1.1)	0.17	0.33
WBC count (G/L)	7.7 (6.5-10.1)	8.3 (6.6-10.2)	8.1 (6.5-10.2)	0.066 [3]	0.25 [3]
Hemoglobin (g/L)	140 (17)	138 (15)	136 (17)	0.46	0.69
Creatinine (μm/L)	90.0 (24.6)	87.2 (22.3)	88.6 (25.5)	0.17	0.21

New neurological deficit					
Admission NIHSS	12.2 (8.2)	13.4 (6.9)	14.3 (5.9)	0.005	0.003
Visual field defects	85 (46.7)	110 (60.1)	125 (69.1)	0.22	0.37
Eye deviation	61 (33.5)	75 (41.0)	95 (52.5)	0.083	0.054
Aphasia	98 (54.1)	90 (48.9)	82 (45.1)	0.052	0.074
Neglect	55 (30.6)[3]	90 (48.9)	99 (55.3)	0.029	0.015
Vigilance impairment	28 (15.6)[3]	22 (12.0)	25 (14.1)	0.005	0.002
Dysarthria	98 (53.9)	108 (59.0)	134 (73.6)	0.58	0.31
Paresis	158 (86.8)	172 (94.0)	176 (96.2)	0.25	0.47
Sensory loss	116 (63.7)	147 (80.8)	148 (80.9)	0.45	0.18
PCA and/or ACA involved	21 (11.5)	15 (8.2)	8 (4.4)	<0.001	<0.001
Onset-to-door time (minutes)	146 (72-441)	128 (82-296)	121 (69-204)	0.22 [3]	0.61 [3]
Onset-to-CT time (minutes)	193 (109-502)	176 (105-403)	141 (96-245)	0.066 [3]	0.40 [3]
Neuroimaging data					
Baseline penumbra	16.5 (5.0 to 35.0)	57.1 (35.8 to 82.3)	117 (92 to 160)	<0.001	<0.001
Early ischemic changes	94 (51.4)	109 (59.2)	100 (54.4)	<0.001	0.004
ASPECTS	9 (6-10)	8 (5-10)	8 (6-10)	<0.001	0.004
Old infarct	55 (30.1)	30 (16.3)	41 (22.3)	0.28	0.042
Leukoaraiosis	44 (24.0)	24 (13.0)	30 (16.3)	0.006	0.002
Hyperdense MCA	53 (34.4)	64 (37.9)	85 (47.2)	0.003	0.003
Clot burden score	7 (1-9)	7 (4-9)	6 (4-8)	<0.001	<0.001
Arterial stenosis ≥50% or occlusion	113 (62.1)	154 (83.7)	181 (98.4)	0.46	0.16
Extracranial stenosis ≥50% or occlusion	49 (26.9)	52 (28.3)	69 (37.5)	0.042	0.067
Intracranial stenosis ≥50% or occlusion	99 (54.4)	146 (79.4)	169 (91.9)	0.65	0.53
Good collaterals	125 (68.3)	112 (60.9)	115 (62.5)	0.003	0.008
Acute treatment (IVT and/or EVT)	77 (42.1)	76 (41.3)	120 (65.2)	0.14	0.018
**Subacute variables**					
Clinical measurements					
Systolic blood pressure (mmHg)	140 (17)	137 (20)	137 (19)	0.48	0.61
Body temperature (°C)	36.7 (0.7)	36.9 (0.6)	36.9 (0.7)	0.35	0.40
Biological data					

Blood glucose (mmol/L)	5.7 (4.8 to 6.6)	5.6 (4.9 to 6.4)	5.6 (5.1 to 6.5)	0.29 [3]	0.33 [3]
Neuroimaging data					
Recanalization					
No occlusion	77 (49.0)	40 (28.3)	14 (8.5)	<0.001	0.002
Recanalized	42 (26.8)	65 (46.1)	111 (67.3)		
Non-recanalized	38 (24.2)	36 (25.5)	40 (24.2)		
Parenchymal hemorrhage	14 (9.9)	16 (10.7)	15 (8.9)	0.024	0.10
TOAST Mechanism					
Large Artery Atherosclerosis	20 (11.0)	29 (15.9)	34 (18.5)	0.16	0.23
Cardioembolism	74 (40.7)	75 (41.2)	81 (44.0)		
Other determined etiology[1]	32 (17.6)	27 (14.8)	28 (15.2)		
Undetermined etiology[2]	56 (30.8)	51 (28.0)	41 (22.3)		

Values are n(%), mean (SD) or median (IQR), P-values calculated using [1]ordinal or using a [2] multinomial logistic regression models adjusted for baseline penumbra. [3] calculated on log transformed values. Abbreviations: ACA=anterior cerebral artery, ASPECTS=Alberta stroke program early compute tomography score, BMI=body mass index, IQR=interquartile range, MCA=middle cerebral artery, mRS=modified Rankin score, NIHSS=National Institutes of Health Stroke Scale, PCA=posterior cerebral artery, SD=standard deviation, TOAST=Trial of Org 10172 in Acute Stroke Treatment, WBC= white blood cells [1] included small vessel disease, dissection and other rare causes. [2] included unknow etiologies or multiple causes.

Table S2. Univariate association of infarct growth with acute and subacute characteristics.

	**24-hour change in Infarct core**				
	**<33th percentile**	**33-<66th percentiles**	**≥ 66th percentiles**		
Median (range)	-25.7 (-50.0 to -7.3)	-0.0 (-7.2 to 8.4)	35.0 (18.4 to 250.0)		
Number of patients	183	184	184	P-Value[1]	P-Value[2]
**Acute Variables**					
Demographics					
Age (yrs)	65.8 (13.1)	66.8 (15.6)	66.3 (15.5)	0.89	0.95
Males	100 (54.6)	97 (52.7)	83 (45.1)	0.18	0.022
Medical history					
Hypertension	109 (59.6)	117 (63.6)	114 (62.0)	0.69	0.90
Dyslipidemia	108 (59.0)	127 (69.4)	125 (68.3)	0.11	0.30
Diabetes	25 (13.7)	22 (12.0)	35 (19.0)	0.10	0.23
Current smoking	40 (21.9)	49 (26.9)	59 (32.4)	0.023	0.080
Previous stroke or TIA	26 (14.2)	40 (21.7)	37 (20.1)	0.34	0.54
Atrial Fibrillation	73 (40.0)	43 (23.4)	69 (37.7)	0.97	0.028
Treatment before stroke					
Lipid-lowering drug	38 (20.8)	42 (22.8)	42 (22.8)	0.61	0.85

Anti-hypertensive drug	97 (53.3)	91 (49.7)	94 (51.7)	0.82	0.88
Anti-diabetic drug	16 (8.7)	9 (4.9)	23 (12.5)	0.12	0.067
Antiplatelet	47 (25.7)	63 (34.2)	61 (33.2)	0.16	0.25
Anticoagulant	22 (12.0)	13 (7.1)	17 (9.2)	0.52	0.86
Pre-stroke mRS	45 (24.6)	53 (28.8)	57 (31.0)	0.27	0.38
Clinical measurements					
Body temperature (°C)	36.3 (0.7)	36.3 (0.7)	36.3 (0.7)	0.70	0.43
Systolic Blood Pressure (mmHg)	153 (26)	156 (26)	155 (31)	0.74	0.96
BMI (kg/m^2)	26.6 (4.8)	25.1 (4.1)	25.2 (4.8)	0.045	0.13
Biological data					
Blood glucose (mmol/L)	6.5 (5.8-7.6)	6.4 (5.7-7.1)	7.0 (6.0-8.6)	<0.001[3]	0.001[3]
Total cholesterol (mmo/L)	5.2 (1.2)	5.4 (1.2)	5.3 (1.2)	0.91	0.82
WBC count (G/L)	7.9 (6.4-9.8)	7.6 (6.5-10.1)	8.5 (6.8-10.3)	0.080[3]	0.20[3]
Hemoglobin (g/L)	139 (15)	139 (16)	136 (18)	0.055	0.13
Creatinine (μm/L)	90.5 (26.1)	88.0 (20.6)	87.4 (25.4)	0.37	0.41
New neurological deficit					
Admission NIHSS	13.8 (6.4)	10.5 (6.8)	15.6 (7.2)	<0.001	<0.001
Visual field defects	119 (65.8)	69 (38.1)	132 (71.7)	0.002	0.001
Eye deviation	83 (45.9)	47 (26.0)	101 (54.9)	<0.001	0.001
Aphasia	92 (50.6)	83 (45.6)	95 (51.9)	0.71	0.55
Neglect	94 (51.7)	66 (36.7)	84 (46.4)	0.88	0.95
Vigilance impairment	21 (11.7)	13 (7.3)	41 (22.7)	<0.001	<0.001
Dysarthria	115 (63.5)	89 (48.9)	136 (73.9)	0.004	0.003
Paresis	170 (93.9)	161 (88.0)	175 (95.1)	0.37	0.41
Sensory loss	138 (76.7)	121 (66.1)	152 (82.6)	0.021	0.016
PCA and/or ACA involved	5 (3.8)	5 (2.7)	32 (17.4)	<0.001	<0.001
Onset-to-door time (minutes)	110 (72-184)	159 (91-515)	132 (66-289)	0.49[3]	0.080[3]
Onset-to-CT time (minutes)	145 (105-222)	203 (120-500)	173 (92-385)	0.54[3]	0.52[3]
Neuroimaging data					
Baseline infarct core	48.2 (27.7 to 89.3)	8 (1.3 to 25.7)	40.4 (10.2 to 91.3)	<0.001[3]	<0.001[3]
Early ischemic changes	86 (47.0)	92 (50.0)	125 (68.0)	<0.001	<0.001
ASPECTS	8 (6-10)	9 (7-10)	7 (5-10)	<0.001	<0.001
Old infarct	31 (16.9)	45 (24.5)	50 (27.2)	0.039	0.098
Leukoaraiosis	24 (11.1)	30 (16.3)	44 (23.9)	0.008	0.023
Hyperdense MCA	66 (37.9)	41 (26.6)	95 (54.3)	<0.001	<0.001
Clot burden score	7 (4-9)	7 (5-9)	4 (1-8)	<0.001	<0.001

Arterial stenosis≥50% or occlusion	163 (89.1)	122 (66.7)	163 (88.6)	0.13	0.18
Extracranial stenosis≥50% or occlusion	42 (23.0)	47 (25.7)	81 (44.0)	<0.001	<0.001
Intracranial stenosis≥50% or occlusion	183 (84.7)	110 (60.1)	149 (81.0)	0.42	0.70
Good Collaterals	117 (63.9)	141 (76.6)	94 (51.1)	<0.001	<0.001
Acute treatment (IVT and/or EVT)	109 (59.6)	68 (37.0)	96 (52.2)	0.52	0.20
**Subacute variables**					
Clinical measurements					
Systolic Blood Pressure (mmHg)	137 (19)	136 (21)	140 (21)	0.13	0.12
Body temperature (°C)	36.9 (0.7)	36.8 (0.6)	36.9 (0.7)	0.58	0.79
Biological data					
Blood glucose (mmol/L)	5.5 (4.9-6.5)	5.4 (4.9-6.4)	5.9 (5.1-6.7)	0.002[3]	0.006[3]
Neuroimaging data					
Recanalization					
No occlusion	27 (16.6)	72 (47.1)	32 (21.8)	<0.001	<0.001
Recanalized	107 (65.6)	52 (34.0)	59 (40.1)		
Non-recanalized	29 (17.8)	29 (19.0)	56 (38.1)		
Parenchymal hemorrhage	11 (6.8)	9 (6.6)	26 (15.6)	0.001	0.012
TOAST Mechanism					
Large Artery Atherosclerosis	20 (11.0)	32 (17.4)	31 (17.0)	0.25	0.34
Cardioembolism	91 (50.0)	65 (35.3)	74 (40.7)		
Other detremined etiology[1]	25 (13.7)	31 (16.9)	31 (17.0)		
Undetermined etiology[2]	46 (25.3)	56 (30.4)	46 (25.3)		

Values are n(%), mean (SD) or median (IQR). P-values calculated using [1]ordinal or using a [2] multinomial logistic regression models adjusted for baseline infarct core. [3] calculated on log transformed values. Abbreviations: ACA=anterior cerebral artery, ASPECTS=Alberta stroke program early compute tomography score, BMI=body mass index, IQR=interquartile range, MCA=middle cerebral artery, mRS=modified Rankin score, NIHSS=National Institutes of Health Stroke Scale, PCA=posterior cerebral artery, SD=standard deviation, TOAST=Trial of Org 10172 in Acute Stroke Treatment, WBC= white blood cells [1] included small vessel disease, dissection and other rare causes. [2] included unknow etiologies or multiple causes.

Table S3. Association of Penumbra Salvage with 12-month outcomes (overall degree of disability, excellent outcome, favorable outcome, and all-cause mortality).

	24-hour volume change in penumbra, ml

	<33th percentile	33-66th percentiles	≥ 66th percentiles	
Median (range)	2.6 (-50.0 to 26.7)	59.3 (26.8 to 97.6)	139.8 (97.7 to 250.0)	
Number of patients	183	184	184	P-Value[1]
mRs (shift analysis)				
median (IQR)	2 (1-6)	2 (1-4)	2 (1-3)	
Baseline-penumbra adjusted OR (95%CI) [2]	1.00 (reference)	2.45 (1.59 to 3.77)	5.47 (3.24 to 9.21)	<0.0001
Fully-adjusted OR (95%CI) [3]	1.00 (reference)	1.66 (1.04 to 2.63)	3.15 (1.78 to 5.57)	<0.0001
c-mRs 0-1				
Baseline-penumbra adjusted rates, %	19.4	41.1	55.4	
Baseline-penumbra adjusted OR (95%CI)	1.00 (reference)	2.89 (1.65 to 5.05)	5.15 (2.61 to 10.15)	<0.0001
Fully-adjusted OR (95%CI) [3]	1.00 (reference)	2.27 (1.15 to 4.50)	4.04 (1.72 to 9.48)	0.002
c-mRs 0-2				
Baseline-penumbra adjusted rates, %	37.7	54.5	72.9	
Baseline-penumbra adjusted OR (95%CI)	1.00 (reference)	1.98 (1.18 to 3.29)	4.44 (2.38 to 8.26)	<0.0001
Fully-adjusted OR (95%CI) [3]	1.00 (reference)	1.22 (0.66 to 2.24)	2.47 (1.18 to 5.16)	0.009
All-cause mortality				
n(%)	39.4	15.7	5.6	
Baseline-penumbra adjusted OR (95%CI)	1.00 (reference)	0.29 (0.16 to 0.51)	0.09 (0.04 to 0.20)	<0.0001
Fully-adjusted OR (95%CI) [3]	1.00 (reference)	0.39 (0.20 to 0.77)	0.16 (0.06 to 0.39)	<0.0001

[1] P-values calculated by including the tertiles of volume change in penumbra as an ordinal variable into logistic regression models. [2] common OR for 1-point improvement in mRs. [3] adjusted for predictors of penumbra salvage (baseline penumbra, leukoaraiosis, PCA and/or ACA involved, neglect, early ischemic changes, clot burden score, BMI, WBC, recanalization status and parenchymal hemorrhage at 24hours) (calculated after handling missing data by multiple imputation procedure (m=10 imputations). Abbreviations: ACA=anterior cerebral artery, BMI=body mass index, CI=confidence interval, c-mRs=corrected modified rankin score, mRs=modified Rankin score, OR=odds ratio, PCA=posterior cerebral artery, WBC= white blood cells.

Table S4. Association of Infarct Growth with 12-month outcomes (overall degree of disability, excellent outcome, favorable outcome, and all-cause mortality).

	24-hours change in infarct core, ml			
	<33th percentile	33-<66th percentiles	≥ 66th percentiles	
Median (range)	-25.7 (-50.0 to -7.3)	-0.0 (-7.2 to 8.4)	35.0 (18.4 to 250.0)	
Number of patients	183	184	184	P-Value[1]
mRs (shift analysis)				
median (IQR)	2 (1-3)	2 (1-3)	4 (2-6)	
Baseline-infarct core adjusted OR (95%CI) [2]	1.00 (reference)	0.50 (0.32 to 0.76)	0.17 (0.11 to 0.25)	<0.0001

Fully-adjusted OR (95%CI) [3]	1.00 (reference)	0.74 (0.47 to 1.16)	0.32 (0.20 to 0.50)	<0.0001
c-mRs 0-1				
Baseline-infarct core adjusted rates, %	55.6	38.6	16.7	
Baseline-infarct core adjusted OR (95%CI)	1.00 (reference)	0.50 (0.30 to 0.84)	0.16 (0.09 to 0.28)	<0.0001
Fully-adjusted OR (95%CI) [3]	1.00 (reference)	0.91 (0.50 to 1.66)	0.35 (0.18 to 0.65)	0.0006
c-mRs 0-2				
Baseline-infarct core adjusted rates, %	0.74	0.58	0.33	
Baseline-infarct core adjusted OR (95%CI)	1.00 (reference)	0.49 (0.29 to 0.82)	0.18 (0.11 to 0.29)	<0.0001
Fully-adjusted OR (95%CI) [3]	1.00 (reference)	0.68 (0.38 to 1.22)	0.35 (0.20 to 0.62)	0.0002
All-cause mortality				
Baseline-infarct core adjusted rates, %	6.3	14.3	30.1	
Baseline-infarct core adjusted OR (95%CI)	1.00 (reference)	2.49 (1.20 to 5.14)	6.44 (3.53 to 11.74)	<0.0001
Fully-adjusted OR (95%CI) [3]	1.00 (reference)	2.26 (1.02 to 5.01)	4.27 (2.12 to 8.59)	<0.0001

[1] P-values calculated by including the tertiles of volume change in infarct core as an ordinal variable into logistic regression models. [2] common OR for 1-point improvement in mRs. [3] adjusted for predictors of infarct growth (baseline infarct core, early ischemic changes, PCA and/or ACA involved, acute blood glucose, leukoaraiosis, hyperdense MCA, extracranial stenosis ≥50% or occlusion, clot burden score, current smoking, old infarct, recanalization status, and parenchymal hemorrhage at 24hours) (calculated after handling missing data by multiple imputation procedure (m=10 imputations). Abbreviations: ACA=anterior cerebral artery, CI=confidence interval, c-mRs=corrected modified rankin score, MCA=middle cerebral artery, mRs=modified rankin score, OR=odds ratio, PCA=posterior cerebral artery.

SUPPLEMENTAL FIGURES.

Supplemental figure S1 A and B. Results of multivariate analysis assessing of penumbra salvage categorized into 3-level categorical variable according to tertiles of 24-hours volume change.

A) Model 1 with acute predictors

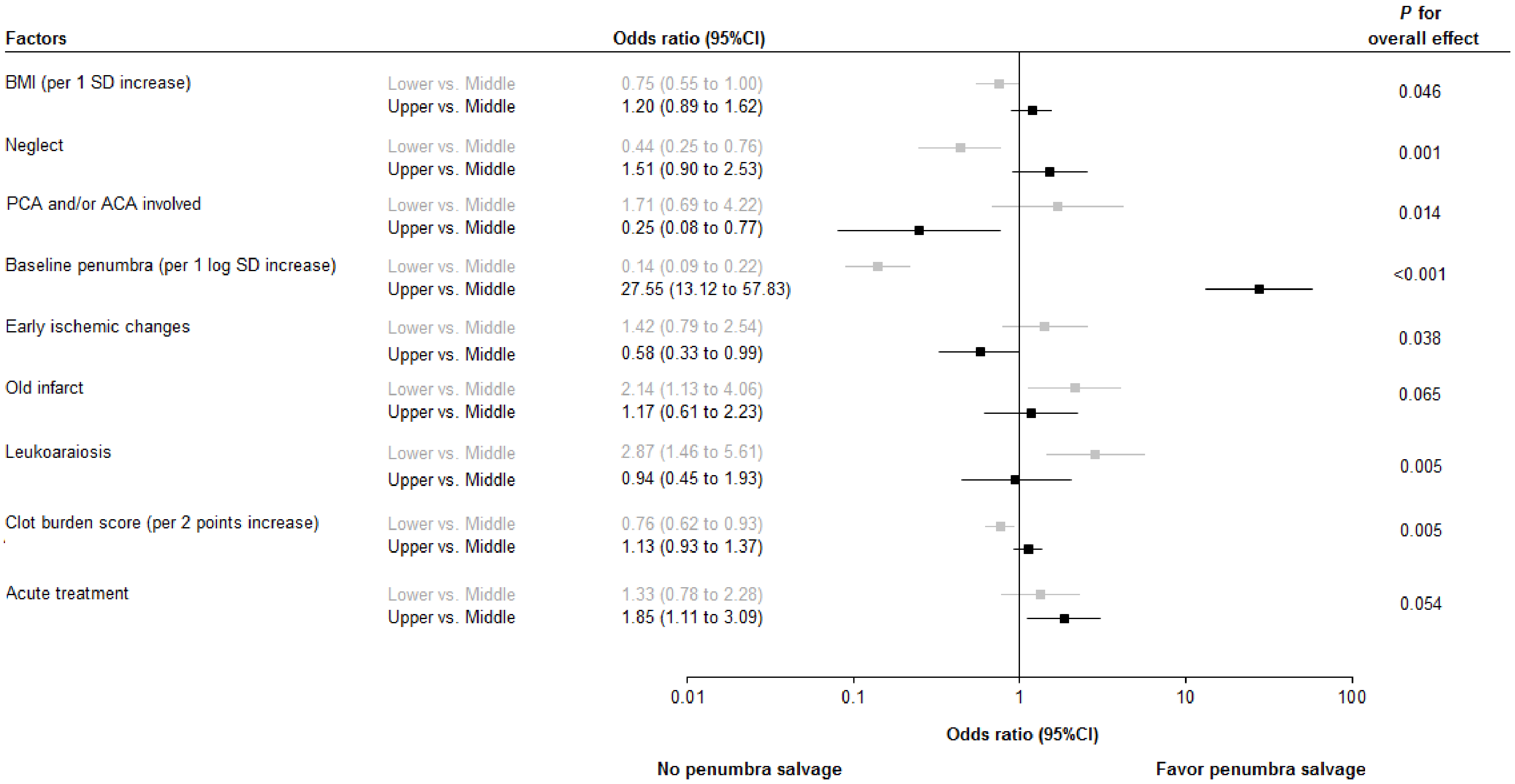

B) Model 2 with acute and subacute predictors

Factors		Odds ratio (95%CI)	*P* for overall effect
BMI (per 1 SD increase)	Lower vs. Middle	0.74 (0.54 to 1.00)	0.042
	Upper vs. Middle	1.22 (0.90 to 1.65)	
Neglect	Lower vs. Middle	0.46 (0.26 to 0.80)	0.004
	Upper vs. Middle	1.43 (0.85 to 2.42)	
PCA and/or ACA involved	Lower vs. Middle	1.77 (0.72 to 4.36)	0.019
	Upper vs. Middle	0.27 (0.08 to 0.83)	
Baseline penumbra (per 1 log SD increase)	Lower vs. Middle	0.14 (0.09 to 0.23)	<0.001
	Upper vs. Middle	28.98 (13.69 to 61.33)	
Early ischemic changes	Lower vs. Middle	1.38 (0.76 to 2.48)	0.025
	Upper vs. Middle	0.53 (0.30 to 0.92)	
Old infarct	Lower vs. Middle	2.20 (1.15 to 4.18)	0.053
	Upper vs. Middle	1.33 (0.68 to 2.59)	
Leukoaraiosis	Lower vs. Middle	2.82 (1.44 to 5.55)	0.007
	Upper vs. Middle	0.93 (0.44 to 1.93)	
Clot burden score (per 2 points increase)	Lower vs. Middle	0.75 (0.60 to 0.93)	0.003
	Upper vs. Middle	1.18 (0.96 to 1.44)	
Recanalisation			0.015
Recanalized vs. no occlusion	Lower vs. Middle	0.76 (0.34 to 1.67)	*0.034*
	Upper vs. Middle	3.54 (1.38 to 9.10)	
Not Recanalized vs. no occlusion	Lower vs. Middle	1.17 (0.50 to 2.75)	*0.56*
	Upper vs. Middle	1.90 (0.69 to 5.20)	

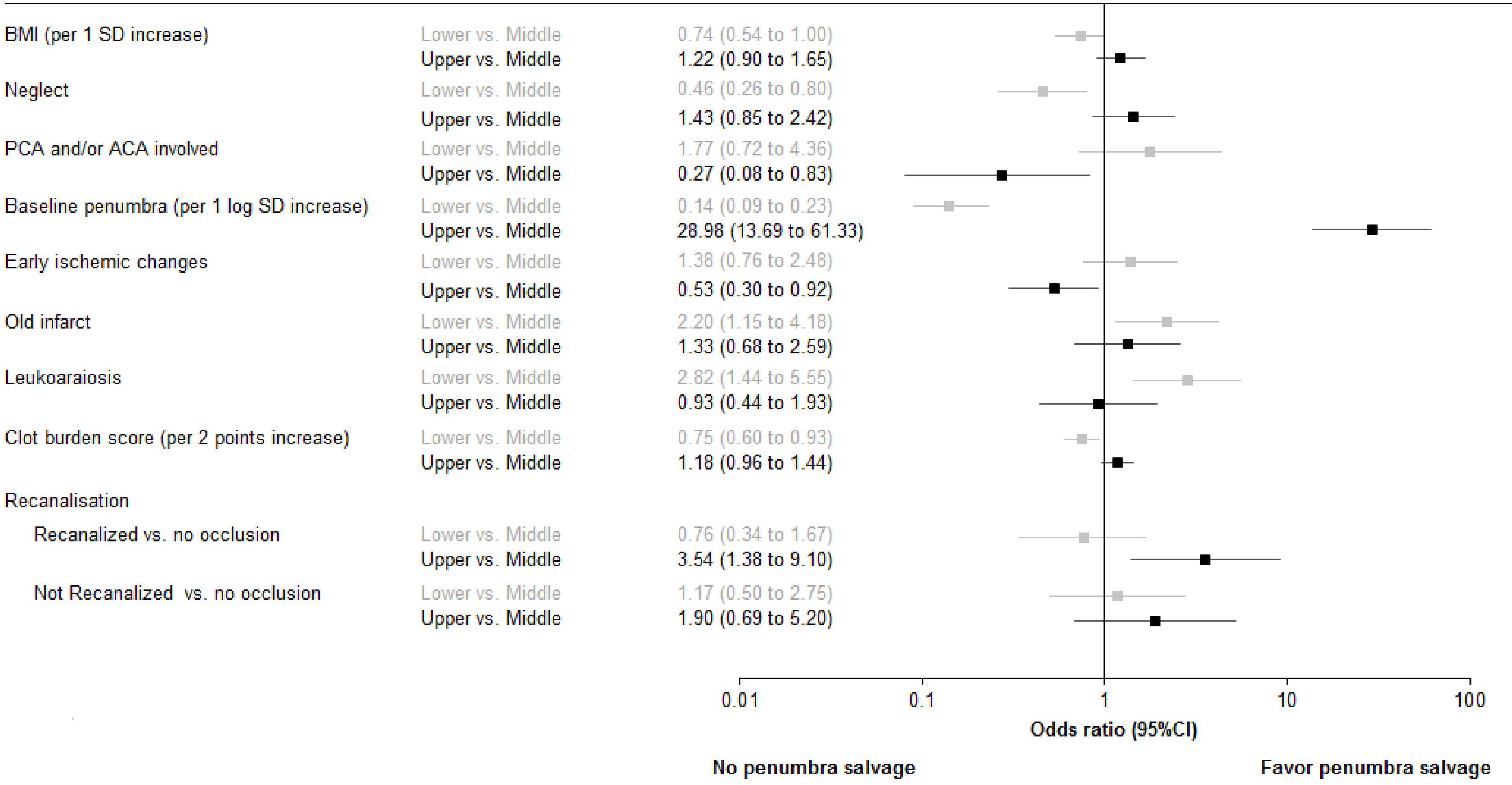

Odds ratios were computed using a backward-stepwise multinomial logistic regression analysis (using middle tertile of penumbra salvage as reference category) after handling missing data by multiple imputation procedure (m=10 imputations). Model 1 included all univariate acute predictors at P<0.10 (except admission NIHSS score since individual components were candidates). Model 2 included all univariate acute and subacute predictors at P<0.10 (except admission NIHSS score since individual components were candidates). Abbreviation: ACA=anterior cerebral artery, BMI=body mass index, NIHSS=National Institutes of Health Stroke Scale, PCA=posterior cerebral artery, SD=standard deviation, WBC= white blood cells.

Supplemental figure S2 A and B. Results of multivariate analysis assessing predictors of infarct growth categorized into 3-level categorical variable according to tertiles of change in infarct core.

A) Model 1 with acute predictors

Factors		Odds ratio (95%CI)	*P* for overall effect
Men	Lower vs. Middle	0.49 (0.28 to 0.85)	0.005
	Upper vs. Middle	0.46 (0.28 to 0.76)	
Current smoking	Lower vs. Middle	1.04 (0.56 to 1.91)	0.085
	Upper vs. Middle	1.72 (1.00 to 2.96)	
Atrial fibrillation	Lower vs. Middle	1.91 (1.07 to 3.42)	0.067
	Upper vs. Middle	1.66 (0.97 to 2.83)	
Glucose (per 1 log SD increase)	Lower vs. Middle	0.91 (0.68 to 1.20)	<0.001
	Upper vs. Middle	1.43 (1.11 to 1.83)	
PCA and/or ACA involved	Lower vs. Middle	0.73 (0.18 to 2.86)	<0.001
	Upper vs. Middle	6.24 (2.13 to 18.27)	
Baseline infarct core (per 1 log SD increase)	Lower vs. Middle	14.38 (8.52 to 24.26)	<0.001
	Upper vs. Middle	1.90 (1.36 to 2.67)	
Early ischemic changes	Lower vs. Middle	0.21 (0.12 to 0.39)	<0.001
	Upper vs. Middle	0.88 (0.51 to 1.52)	
Old infarct	Lower vs. Middle	0.69 (0.35 to 1.33)	0.084
	Upper vs. Middle	1.38 (0.79 to 2.41)	
Leukoaraiosis	Lower vs. Middle	0.67 (0.32 to 1.42)	0.011
	Upper vs. Middle	1.84 (1.00 to 3.39)	
Hyperdense MCA	Lower vs. Middle	0.80 (0.40 to 1.57)	0.046
	Upper vs. Middle	1.71 (0.90 to 3.23)	
Clot burden score (per 2 point increase)	Lower vs. Middle	1.86 (1.03 to 3.36)	0.014
	Upper vs. Middle	1.19 (0.64 to 2.18)	
Extracranial stenosis≥50% or occlusion	Lower vs. Middle	0.80 (0.42 to 1.51)	0.032
	Upper vs. Middle	1.65 (0.95 to 2.87)	
Good collaterals	Lower vs. Middle	1.93 (1.01 to 3.68)	0.066
	Upper vs. Middle	1.03 (0.57 to 1.84)	

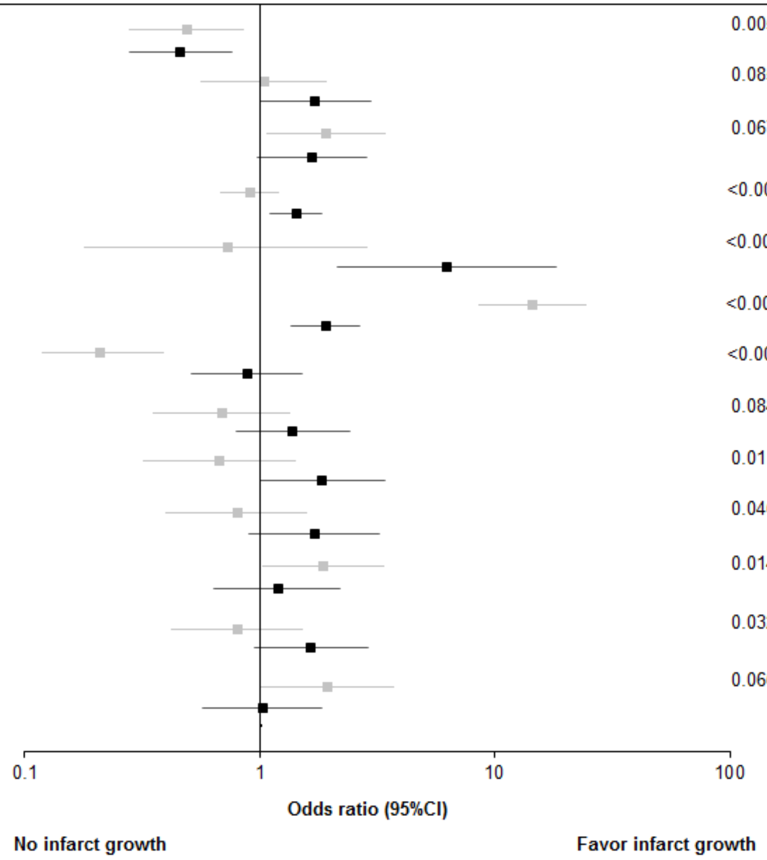

B) Model 2 with acute and subacute predictors

Factors		Odds ratio (95%CI)	*P* for overall effect
Men	Lower vs. Middle	0.45 (0.26 to 0.73)	0.003
	Upper vs. Middle	0.44 (0.26 to 0.73)	
Anti-diabetic drug	Lower vs. Middle	1.99 (0.64 to 6.18)	0.089
	Upper vs. Middle	3.07 (1.12 to 8.38)	
Glucose (per 1 log SD increase)	Lower vs. Middle	0.91 (0.68 to 1.20)	0.091
	Upper vs. Middle	1.43 (1.11 to 1.83)	
PCA and/or ACA involved	Lower vs. Middle	0.77 (0.19 to 3.10)	<0.001
	Upper vs. Middle	5.71 (1.93 to 16.93)	
Baseline infarct core (per 1 log SD increase)	Lower vs. Middle	15.29 (8.78 to 26.61)	<0.001
	Upper vs. Middle	1.81 (1.29 to 2.55)	
Early ischemic changes	Lower vs. Middle	0.20 (0.11 to 0.38)	<0.001
	Upper vs. Middle	0.85 (0.48 to 1.47)	
Old infarct	Lower vs. Middle	0.62 (0.31 to 1.21)	0.071
	Upper vs. Middle	1.31 (0.75 to 2.31)	
Leukoaraiosis	Lower vs. Middle	0.78 (0.36 to 1.65)	0.017
	Upper vs. Middle	1.94 (1.05 to 3.57)	
Hyperdense MCA	Lower vs. Middle	0.77 (0.37 to 1.55)	0.017
	Upper vs. Middle	1.88 (0.98 to 3.61)	
Clot burden score (per 2 point increase)	Lower vs. Middle	1.38 (1.05 to 1.82)	0.006
	Upper vs. Middle	0.92 (0.71 to 1.17)	
Extracranial stenosis≥50% or occlusion	Lower vs. Middle	0.78 (0.40 to 1.51)	0.063
	Upper vs. Middle	1.57 (0.88 to 2.77)	
Good collaterals	Lower vs. Middle	1.94 (1.01 to 3.70)	0.059
	Upper vs. Middle	0.99 (0.54 to 1.80)	
Recanalisation			0.008
Recanalized vs. no occlusion	Lower vs. Middle	2.62 (1.18 to 5.81)	*0.029*
	Upper vs. Middle	0.96 (0.46 to 1.99)	
Not Recanalized vs. no occlusion	Lower vs. Middle	1.48 (0.57 to 3.81)	*0.56*
	Upper vs. Middle	1.47 (0.70 to 3.06)	
Parenchymal hematoma	Lower vs. Middle	0.46 (0.13 to 1.58)	0.051
	Upper vs. Middle	1.58 (0.63 to 3.99)	

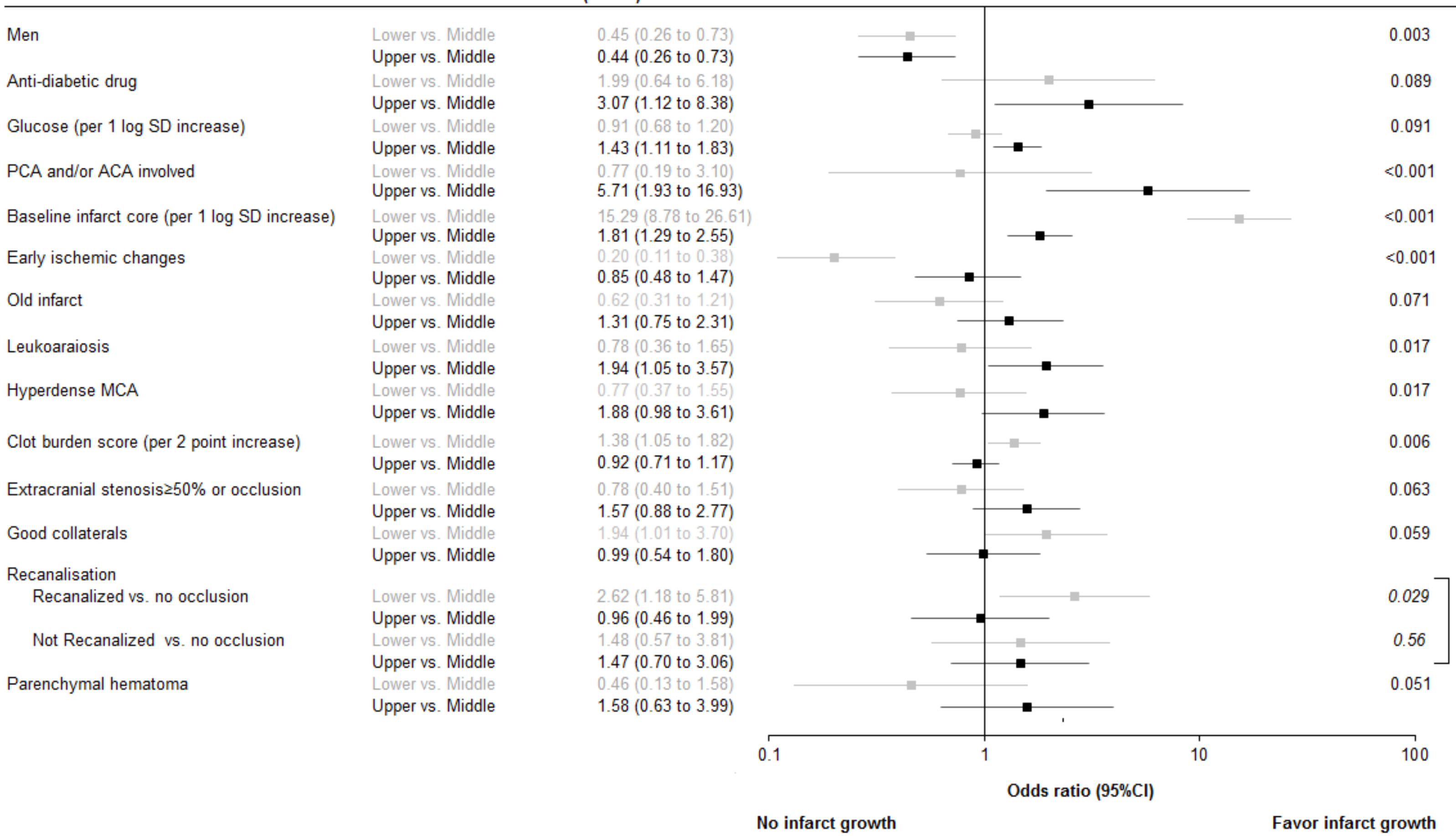

Odds ratios were computed using a backward-stepwise multinomial logistic regression analysis (using middle tertile of change in infarct core as reference category) after handling missing data by multiple imputation procedure (m=10 imputations). Model 1 included all univariate acute predictors at P<0.10 (except admission NIHSS score since individual components were candidates). Model 2 included all univariate acute and subacute predictors at P<0.10 (except admission NIHSS score since individual components were candidates). Abbreviation: ACA=anterior cerebral artery, MCA=middle cerebral artery, NIHSS=National Institutes of Health Stroke Scale, PCA=posterior cerebral artery, SD=standard deviation.
